# Selection and Prioritization of Candidate Drug Targets for Amyotrophic Lateral Sclerosis Through a Meta-Analysis Approach

**DOI:** 10.1007/s12031-017-0898-9

**Published:** 2017-02-24

**Authors:** Giovanna Morello, Antonio Gianmaria Spampinato, Francesca Luisa Conforti, Velia D’Agata, Sebastiano Cavallaro

**Affiliations:** 1 Institute of Neurological Sciences (ISN), Italian National Research Council (CNR), Catania and Mangone (CS), Italy; 2 Department of Biomedical and Biotechnological Sciences, Section of Human Anatomy and Histology, University of Catania, Catania, Italy

**Keywords:** ALS, Meta-analysis, SOD1G93A mouse model, Transcriptomics

## Abstract

Amyotrophic lateral sclerosis (ALS) is a progressive and incurable neurodegenerative disease. Although several compounds have shown promising results in preclinical studies, their translation into clinical trials has failed. This clinical failure is likely due to the inadequacy of the animal models that do not sufficiently reflect the human disease. Therefore, it is important to optimize drug target selection by identifying those that overlap in human and mouse pathology. We have recently characterized the transcriptional profiles of motor cortex samples from sporadic ALS (SALS) patients and differentiated these into two subgroups based on differentially expressed genes, which encode 70 potential therapeutic targets. To prioritize drug target selection, we investigated their degree of conservation in superoxide dismutase 1 (SOD1) G93A transgenic mice, the most widely used ALS animal model. Interspecies comparison of our human expression data with those of eight different SOD1^G93A^ datasets present in public repositories revealed the presence of commonly deregulated targets and related biological processes. Moreover, deregulated expression of the majority of our candidate targets occurred at the onset of the disease, offering the possibility to use them for an early and more effective diagnosis and therapy. In addition to highlighting the existence of common key drivers in human and mouse pathology, our study represents the basis for a rational preclinical drug development.

**Electronic supplementary material:**

The online version of this article (doi:10.1007/s12031-017-0898-9) contains supplementary material, which is available to authorized users.

## Background

Amyotrophic lateral sclerosis (ALS), sometimes called Lou Gehrig’s disease, is a fatal neurodegenerative disorder caused by the loss of both upper and lower motoneurons. Approximately 10% of ALS cases is familial (FALS), mainly with an autosomal dominant inheritance pattern, while the majority of cases (90%) occurs sporadically (SALS), caused by complex interaction between multiple genetic variants and environmental factors (Mitchell and Borasio 2007).

Although, over the last years, several studies have been conducted to develop effective therapeutic interventions, ALS remains a progressive and incurable disease (Pasinelli and Brown 2006; Ferraiuolo et al. 2011). In fact, the only approved treatment, riluzole, improves survival of ALS patients but only to a modest extent, whereas several other pharmacologic agents, apparently promising when tested in animal models, have failed when translated into clinical practice (Turner et al. 2001). This may be due to poor knowledge of ALS pathophysiology and the use of inadequate animal models.

During the last 20 years, the most popular animal model of ALS has been the superoxide dismutase 1 G93A transgenic mouse (SOD1^G93A^), overexpressing human *SOD1* with a causative mutation (a glycine to alanine substitution at position 93), which results in a toxic gain of SOD1 function (Dal Canto and Gurney 1994; Achilli et al. 2005). Although the use of this and other animal models (e.g., TDP-43, TAU P301L, and Wobbler mice) has provided invaluable tools for ALS research, they do not faithfully reproduce the complexity and heterogeneity characterizing the human disease. It is evident, therefore, that there is a need to use research strategies that allow to optimize the identification and selection of clinically useful therapeutic targets overcoming the current disconnection between preclinical studies and the translation of these results into clinical practice (Perrin 2014; McGoldrick et al. 2013).

The advent of high-throughput experimental technologies, such as DNA microarray, has revolutionized the field of biological research, becoming one of the major methods to elucidate the transcriptional features of many complex diseases, such as ALS, and to identify/prioritize new pharmacological targets (Lederer et al. 2007a; Morello et al. 2015; Paratore et al. 2006; Cavallaro et al. 2012). To this regard, our research group has recently characterized the transcriptional profiles of motor cortex samples from SALS patients and differentiated these into two gene expression-based subgroups (SALS 1 and SALS2), revealing new clues to the molecular pathogenesis and novel potential predictive biomarkers and therapeutic targets (Aronica et al. 2015; Morello and Cavallaro 2015).

The aim of the present study is to investigate the degree of conservation of the previously identified molecular targets (Aronica et al. 2015; Morello and Cavallaro 2015) in SOD1^G93A^ transgenic mice, in order to prioritize their possible selection for a subsequent validation. The use of a meta-analytic approach offers a unique opportunity to significantly increase the statistical power of any individual microarray study, thus enabling identification of more reliable molecular biomarkers and targets. Moreover, to better characterize the role of candidate target genes in ALS pathophysiology, we have interpreted data in the context of Gene Ontology (GO) annotations and known biological pathways.

## Methods

**Fig. 1:**
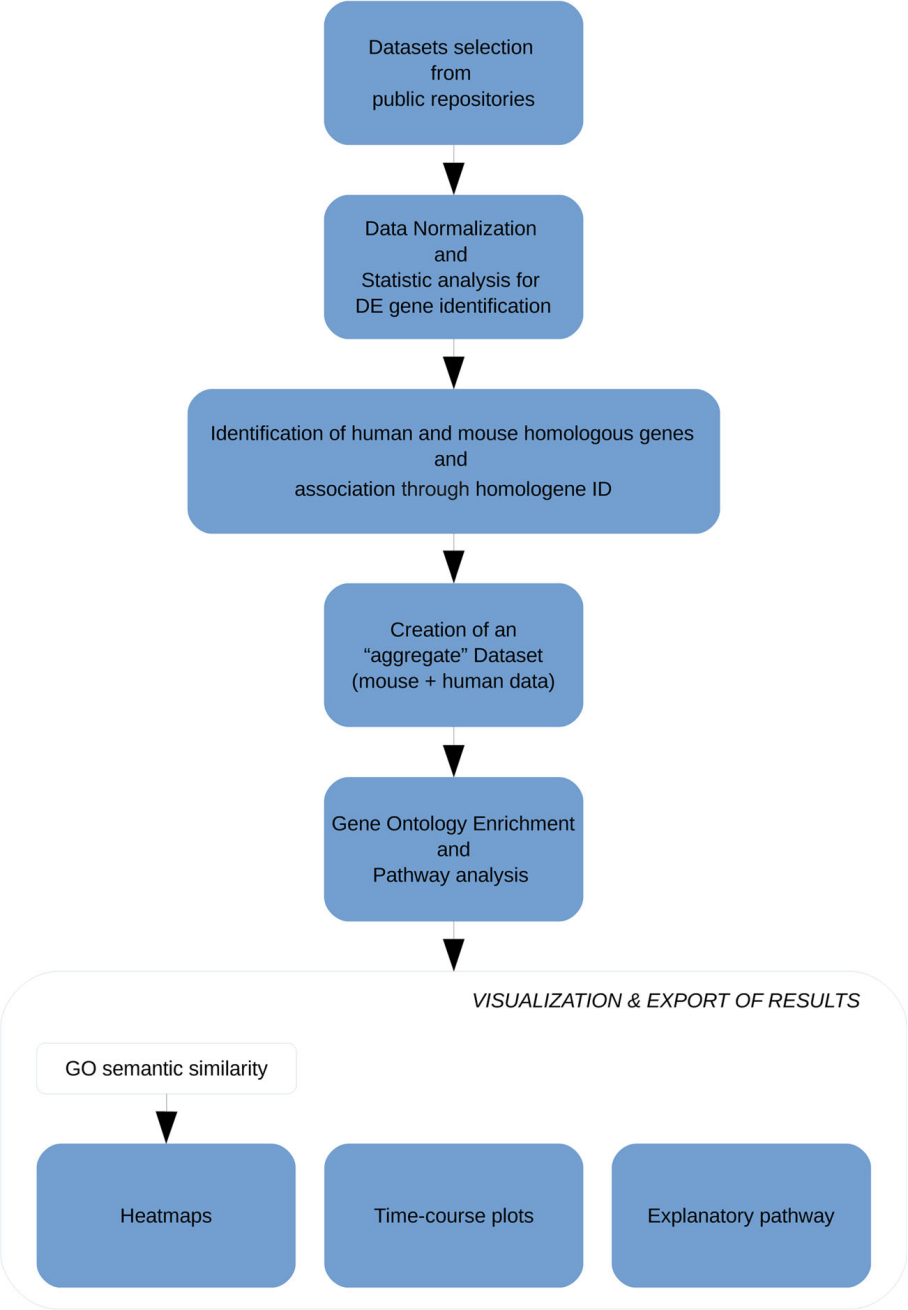
Schematic representation of proposed meta-analysis-based drug target selection and prioritization. The workflow depicts the steps performed in this study, from data acquisition to the visualization and export of results in various output formats. See “Methods” section for details. *DE* differentially expressed, *GO* Gene Ontology

The analysis workflow is shown in Fig. 1 and described below.

### Dataset Selection and Preprocessing

In our previous work (Aronica et al. 2015), we analyzed genes and pathway differentially deregulated in the motor cortex of two subgroups of SALS patients compared with controls. The analysis of altered networks of biological molecules in SALS has enabled to identify genes encoding potential therapeutic targets by combining various drug repositories (e.g., Metacore; Clinical Trials.gov; DrugBank; PubChem). In particular, we selected genes encoding proteins that were primary targets of drugs currently used in preclinical or clinical stages for treating several clinical diseases, giving greater emphasis to those that showed encouraging results for the treatment of neurodegenerative disorders (Aronica et al. 2015; Morello and Cavallaro 2015).

In order to identify common genomic changes between human ALS and SOD1^G93A^ transgenic mouse model, in this work, we compared expression changes of our human gene target list with those of their corresponding homologs in SOD1^G93A^ transgenic mice present in public repositories. To do it, we searched the public data repositories NCBI GEO (http://www.ncbi.nlm.nih.gov/geo/) and ArrayExpress (http://www.ebi.ac.uk/arrayexpress/) for gene expression microarray data sets using the following search terms and/or their combinations: “amyotrophic lateral sclerosis”, “motor neuron disease”, “SOD1 mouse model”, “SOD1G93A transgenic mouse” and “expression profiling”. Data sets included in the analysis had to meet the following criteria: (1) data were acquired using a genome-wide gene expression microarray platform with accessible and clear probe-to-gene mapping annotations and (2) there were ≥3 replicates for each experimental condition. In particular, we utilized only gene expression data obtained from isolated motor neurons and/or spinal cord homogenates from wild-type littermates and SOD1^G93A^ mice at various disease stages (presymptomatic, symptomatic and terminal stage). Overall, for each dataset, the following information were extracted: GEO or ArrayExpress accession number, title, year of publication, reference, platform, number of samples (transgenic mice and controls), stage of disease, sample type and gene expression data (Table 1).

**Table 1:** Overview of public gene expression data sets included in the meta-analysis

Accession no.	Study reference	Year	Title	Platform	Numbers (per stage) and characteristics of patients and mouse models	Total samples	Stage of disease	Tissue
E-MTAB-2325	Aronica et al. (2015)	2014	Gene expression profiles in motor cortex of control and sporadic amyotrophic lateral sclerosis patients	GPL6480: Agilent-014850 Whole Human Genome Microarray 4x44K G4112F	31 SALS patients 10 controls	41	Postmortem	Motor cortex
GSE10953	Ferraiuolo et al. (2007)	2007	Cellular pathways involved in the adaptation and progression of motor neuron injury in the mouse model of familial ALS	GPL339: [MOE430A] Affymetrix Mouse Expression 430A Array	3 SOD1-G93A 3 SOD1-WT	18	60–90-120 days	Total spinal cord (motor neurons isolated using LCM)
GSE56926	Maximino et al. (2014)	2014	Deregulated expression of cytoskeleton related genes in the spinal cord and sciatic nerve of presymptomatic SOD1G93A amyotrophic lateral sclerosis mouse model	GPL13912: Agilent-028005 SurePrint G3 Mouse GE 8x60K Microarray	4 SOD1-G93A 4 non-transgenic controls	8	60 days	Lumbar spinal cord
GSE50642	de Oliveira et al. (2013)	2013	Early gene expression changes in spinal cord from SOD1G93A amyotrophic lateral sclerosis animal model	GPL7202: Agilent-014868 Whole Mouse Genome Microarray 4x44K G4122F	4 SOD1-G93A 4 non-transgenic controls	16	40–80 days	Lumbar spinal cord
GSE27933	Saris et al. (2013)	2013	Gene expression profile of G93A-SOD1 mouse spinal cord, blood, and muscle	GPL6103: Illumina mouseRef-8 v1.1 expression beadchip	8 SOD1-G93A 8 SOD1-WT	32	70–100 days	Lumbar spinal cord

For each mouse dataset, raw intensity values were thresholded to 1, log2-transformed, normalized, and baselined to the median of all samples by using GeneSpring GX v13.1 software package (Agilent Technologies). Affymetrix gene expression data were Robust Multichip Average (RMA) processed using quantile normalization, while Agilent and Illumina data were normalized to the 75th percentile (Cavallaro et al. 2002; Cavallaro et al. 2004).

### Meta-Analysis of Gene Expression Data

To perform interspecies comparison, we built a unique, comprehensive dataset where human and mouse gene expression data were aggregated. In particular, this dataset included expression changes of 70 differentially expressed genes encoding potential therapeutic targets, previously characterized in SALS patients, together with their corresponding homologs in mouse. To create this dataset, we firstly associated microarray probe IDs from each dataset to corresponding NCBI Entrez human or mouse gene IDs and symbols by using *Homo sapiens* GRCh38 and *Mus musculus* GRCm38.p2 (Maglott et al. 2005). When multiple probes corresponded to the same gene, gene expression values were averaged. Then, fold change values for human (SALS patients versus control) and mouse (SOD1^G93A^ mice versus littermate control groups) were calculated and converted into the negative reciprocal if the fold change was less than 1. Moreover, gene IDs from mouse studies were annotated with the corresponding human homologs, when available, using NCBI HomoloGene database (build version 68) (Wheeler et al. 2008). All the above-described analyses were performed by using homemade functions in PostgreSQL environment (version 9.4.5; http://www.postgresql.org/).

To assess the statistical significance of common genomic changes observed in terminal stages of both human and ALS animal models, statistical analysis of gene expression data from 120-day-old SOD1^G93A^ transgenic mice was performed by using GeneSpring GX v13.1 software package (Agilent Technologies). Genes with a corrected *P* value <0.05 (one-way ANOVA followed by the Benjamin-Hochberg false discovery rate and the Tukey’s post hoc test) were deemed to be statistically significant.

For a graphical representation of fold regulation expression data, heat maps and time-course plots were generated by using the R packages “heatmap.2” and “ggplot2” (R Core Team, 2012; https://www.r-project.org). Two different types of distance-based hierarchical clustering were also performed by using the “hclust” function in R. The first was based on the Euclidean distance of average fold change values represented in linear scale using complete linkage method as parameter (Ward 1963). The second was based on the semantic similarity among the GO terms for biological processes and gene products and was determined by using the GOSemSim R package (Yu et al. 2010).

To interpret the biological significance of selected candidate targets, GO terms and pathways enrichment analyses were performed using Metacore (Thomson Reuters) (Nikolsky et al. 2009). *P* values were obtained from the basic formula for hypergeometric distribution and corrected by a false discovery rate threshold of 0.05.

## Results

### Study Characteristics

We collected and manually curated a total of five expression profile studies in human ALS and SOD1^G93A^ mice, comprising a total of nine datasets (Table 1). In particular, we used human genomic data published in our previous work, where we analyzed whole-genome expression profiles of 41 motor cortex samples of control (10) and sporadic ALS (31) patients. By using an unsupervised hierarchical clustering algorithm, we were able to separate, on the basis of their transcriptome profiles, control from SALS patients, subdividing these latter into two different groups (SALS1 and SALS2), each associated to specific differentially expressed pathways and genes (Aronica et al. 2015). Interestingly, the analysis of altered networks of biological molecules in SALS provided a number of potential therapeutic targets, which could be used to interfere with ALS pathogenesis (Aronica et al. 2015; Morello and Cavallaro 2015).

**Fig. 2:**
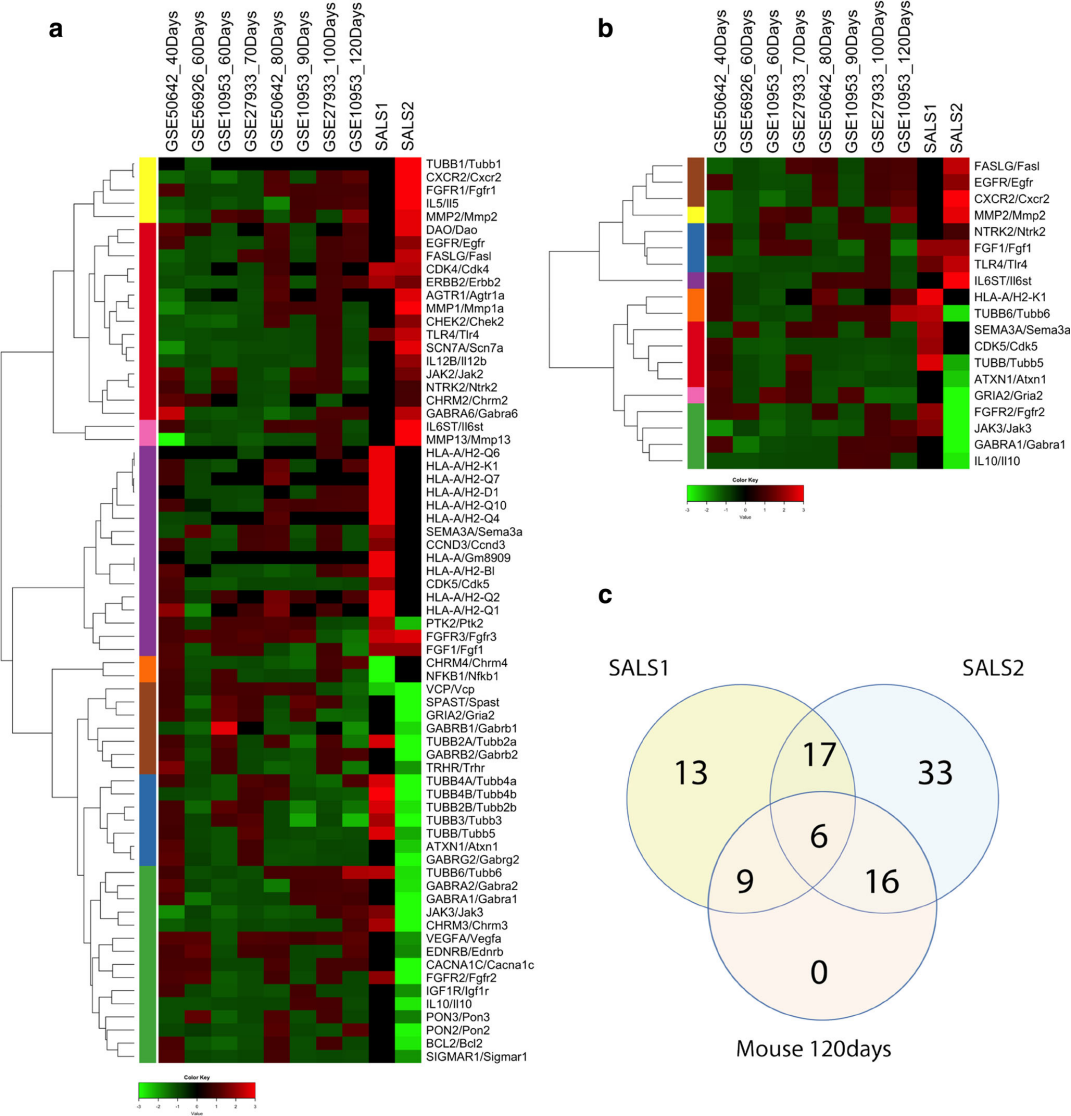
Meta-analysis of gene expression profiles of SOD1^G93A^ mice and SALS patients reveals common candidate therapeutic targets. **a** Hierarchical clustering heat map visualization of the gene expression pattern for 70 promising therapeutic targets across different datasets of SOD1^G93A^ mice (at different ages and stages of disease) and SALS patients. In this two-dimensional presentation, *rows* represent target genes and *columns* denote datasets used in our meta-analysis. From left to right, these datasets included expression profiles of motor neurons from spinal cord of SOD1^G93A^ mice at 40, 60, 70, 80, 90, 100, and 120-day old and motor neurons from motor cortex of two SALS patients’ subgroups. Gene symbols for each human/mouse ortholog pair are shown on the *right hand side* of the picture. Genes were clustered using a hierarchical clustering based on Euclidean distances of average fold change values represented in linear scale and with complete linkage method as parameter. In the dendrograms shown (*left*), the length and the subdivision of the branches display the relatedness of the expression of the genes. Fold change values were calculated as the ratio between SALS patients versus individual controls for the human dataset and between SOD1^G93A^ mice versus littermate control groups for each murine dataset. As shown in the color bar, *red* indicates upregulation, *green* downregulation, and *black* no change. Complete gene list and corresponding fold change values are found in Supplementary Table 1. **b** Hierarchical clustering of 19 statistically significant (*P* value <0.05) differentially expressed target genes commonly deregulated in both end-stage SOD1^G93A^ mice and SALS patients. Genes corresponding to each row of the heat map are on the *right hand side* of the picture. Genes are arranged in a hierarchical cluster based on their expression patterns, combined with a dendrograms (*left*) whose branch lengths reflect the relatedness of expression patterns. As shown in the color bar, *red* indicates upregulation, *green* downregulation, and *black* no change. **c** Venn diagrams of statistically significant differentially expressed target genes in 120-day-old SOD1^G93A^ mice and SALS patient subgroups compared to their corresponding controls (Table 2)

The present analysis focused on the expression changes of 70 target genes previously characterized in SALS patients (33 differentially expressed in SALS1 and 54 in SALS2 patients), together with their corresponding homologs in SOD1^G93A^ mice, using data present in public repositories (Fig. 2a, Supplementary Table 1 and Supplementary Figs. 1a and 2a) (Ferraiuolo et al. 2007; Maximino et al. 2014; de Oliveira et al. 2013; Saris et al. 2013; Kudo et al. 2010). All animal studies were performed on spinal cord samples and included a total of 74 samples (37 from SOD1^G93A^ mice and 37 from littermate control mice). One of these studies applied the technique of laser capture microdissection (LCM) to isolate motor neurons from surrounding tissues, performing a cell-specific analysis for the identification of motor neuron-specific changes (Ferraiuolo et al. 2007; Saris et al. 2013). Another study (GSE56926) was carried out at a single time point (60 days), while others (GSE10953, GSE50642, and GSE27933) included multiple time-course data points. In total, seven time points were used in this study, which can be grouped into three different stages of disease: “pre-symptomatic” (40–60 days), “symptomatic”, characterized by early clinical signs of ALS disease, like muscle tremors (70–90 days) and a “terminal” stage (100–120 days) based on ALS symptom progression. The main features of studies included in this meta-analysis are summarized in Table 1.

### Identification of Commonly Deregulated Drug Targets Both in Terminal Stage SALS Patients and SOD1G93A Mice

After data processing, as indicated in the “Methods” section, an “aggregate dataset” was generated. It includes the expression changes of 70 deregulated genes, encoding potential therapeutic targets previously characterized in SALS patients, together with those of their corresponding homologs in SOD1^G93A^ mouse at different ages and stages of disease. Subsequently, to investigate and select candidate genes consistently conserved between postmortem human samples and end-stage ALS mouse models, we compared expression profiles of these 70 genes in SALS patients with those observed in terminal stage SOD1^G93A^ mice (Fig. 2a, Supplementary Table 1, and Supplementary Figs. 1a and 2a) (Aronica et al. 2015; Morello and Cavallaro 2015). From this comparison, 19 statistically significant genes emerged as deregulated both in mice and SALS patients: 9 commonly altered with SALS1 and 16 with SALS2 patients (Fig. 2b, c, Table **2** , and Supplementary Figs. 1b and 2b). Although some of these genes (7/19) were differentially deregulated between mice and SALS patients, the majority of them (12/19) were deregulated in the same direction in end-stage SOD1^G93A^ mice and in at least one of SALS patient subgroups, suggesting the existence of similar pathogenic mechanisms shared by both species (Table 2).

**Table 2:** List of the 19 candidate targets commonly differentially expressed between SALS patients and end-stage SOD1-G93A mice

**Homolo Gene ID**	**Gene symbol human/mouse**	**Gene name**	**[SALS1 patients] versus [CTRL** ^**+**^ **]** ^**a**^		**[SALS2 patients] versus [CTRL** ^**+**^ **]** ^**a**^		**[SOD1** ^**G93A**^ **_120d] versus [CTRL** ^**+**^ **]** ^**b**^	
			**Fold change**	**Regulation**	**Fold change**	**Regulation**	**Fold change**	**Regulation**
**281**	**ATXN1/Atxn1**	**Ataxin 1**	**–**	**–**	**–2.44**	**Down**	**–1.04**	**Down**
3623	CDK5/Cdk5	Cyclin-dependent kinase 5	2.01	Up	–	–	–1.01	Down
**10439**	**CXCR2/Cxcr2 (IL8RB/Il8rb)**	**Chemokine (C-X-C motif) receptor 2 (interleukin 8 receptor type 2)**	**–**	**–**	**5.02**	**Up**	**1.28**	**Up**
**74545**	**EGFR/Egfr**	**Epidermal growth factor receptor**	**–**	**–**	**1.88**	**Up**	**1.11**	**Up**
533	FASLG/Fasl	Fas ligand (TNF superfamily, member 6)	–	–	2.40	Up	1.09	Up
625	FGF1/Fgf1	Fibroblast growth factor 1	1.92	Up	1.92	Up	–1.6	Down
**22566**	**FGFR2/Fgfr2**	**Fibroblast growth factor receptor 2**	**1.85**	**Up**	**–4.35**	**Down**	**–1.02**	**Down**
629	GABRA1/Gabra1	Gamma-aminobutyric acid (GABA) A receptor, alpha 1	–	–	–4.76	Down	1.02	Up
**20225**	**GRIA2/Gria2**	**Glutamate receptor, ionotropic, AMPA 2**	**–**	**–**	**–3.82**	**Down**	**–1.33**	**Down**
**128352**	**HLA-A/H2-K1**	**Major histocompatibility complex, class I, A**	**2.84**	**Up**	**–**	**–**	**1.19**	**Up**
**478**	**IL10**	**Interleukin 10**	**–**	**–**	**–2.72**	**Down**	**–1.05**	**Down**
1645	IL6ST/Il6st	Interleukin 6 signal transducer (gp130, oncostatin M receptor)	–	–	9.76	Up	–1.08	Down
**181**	**JAK3/Jak3**	**Janus kinase 3**	**1.69**	**Up**	**–3.14**	**Down**	**1.26**	**Up**
**3329**	**MMP2/Mmp2**	**Matrix metallopeptidase 2**	**–**	**–**	**2.79**	**Up**	**1.71**	**Up**
4504	NTRK2(TRKB)/Ntrk2(TrkB)	Neurotrophic tyrosine kinase, receptor, type 2	–	–	1.03	Up	−1	Up
31358	SEMA3A/Sema3a	Sema domain, immunoglobulin domain (Ig), short basic domain, secreted, (semaphorin) 3A	2.13	Up	–	–	–1.08	Down
7099	TLR4/Tlr4	Toll-like receptor 4	–1.03	Down	1.46	Up	2.41	Up
**69099**	**TUBB/Tubb5**	**Tubulin, beta class I**	**2.68**	**Up**	**–2.13**	**Down**	**–1.05**	**Down**
**69414**	**TUBB6/Tubb6**	**Tubulin, beta 6 class V**	**2.30**	**Up**	**–2.62**	**Down**	**2.21**	**Up**

The table shows the overlapping target genes between two subgroups of SALS patients and 120-day-old SOD1^G93A^ mice. For each gene, average fold change values of differential expression between SALS patients versus individual controls and 120-day-old SOD1^G93A^ transgenic mice versus littermate controls are shown. Official Entrez Gene Symbols and Names are used. Bold values indicate the most promising candidate targets that are deregulated in the same direction both in 120-day-old SOD1^G93A^ mice that in at least one of SALS patients subgroups. For the full list of target genes analyzed in this study, see Supplementary Table 1

^a^Aronica et al. (2015) and Paratore et al. (2006)

^b^Ferraiuolo et al. (2007) and Ward (1963)

### Time-Course Analysis of Commonly Deregulated Drug Target Genes in SALS Patients and SOD1^G93A^ Mice at Different Stages of Disease

**Fig. 3:**
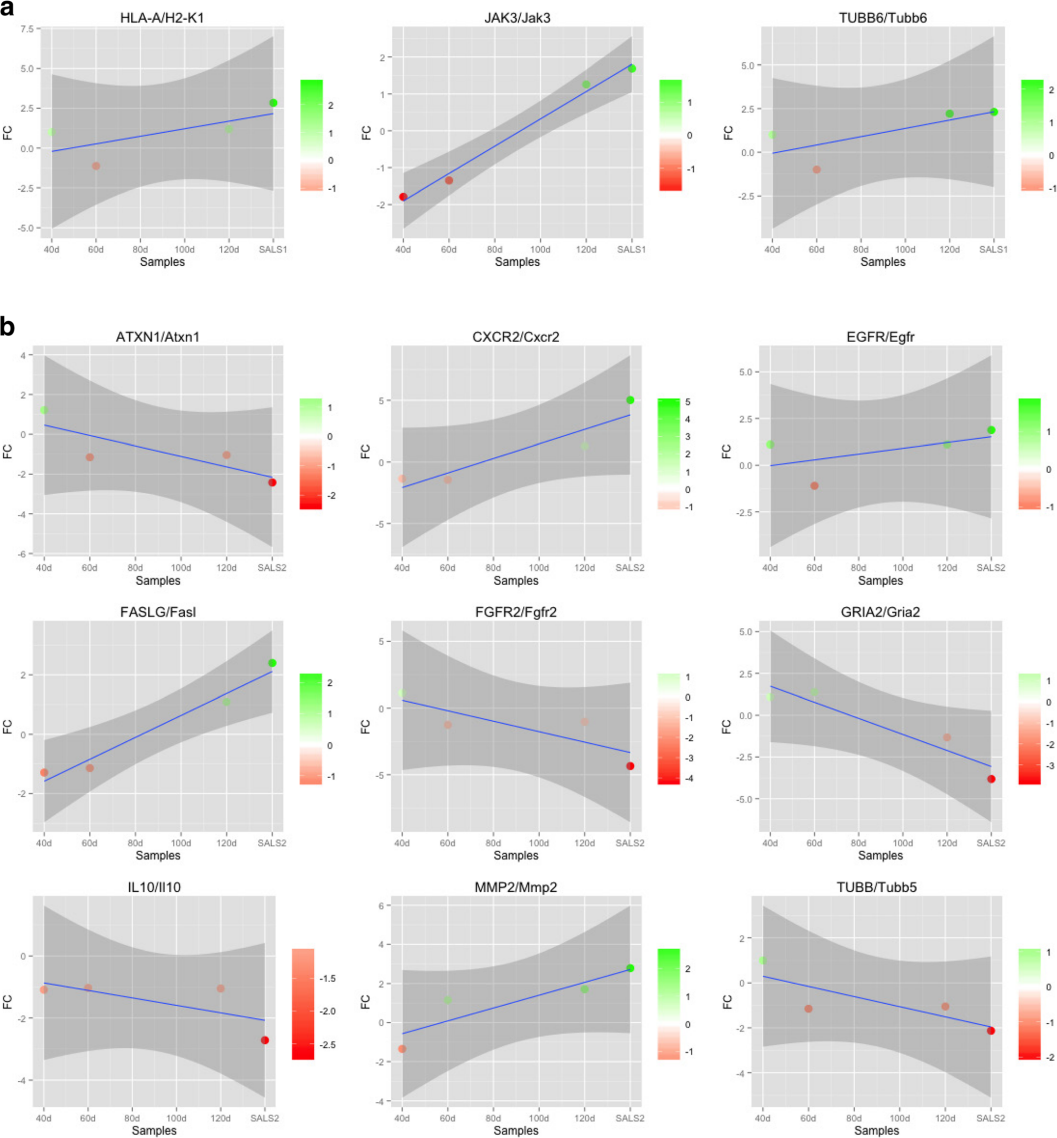
Time-course analysis of commonly deregulated genes in both SALS patients and SOD1^G93A^ mice at different stages of disease. Plots illustrate the correlation of expression fold changes (*y*-axis) of target genes between SALS1 patients (**a**) and SALS2 patients (**b**) versus individual controls and SOD1^G93A^ mice versus littermate controls at 40, 60, 100, and 120 days of age (*x*-axis). For generating time-course analysis of SOD1^G93A^ mice, we referred to following datasets: GSE10953 for 60, 90, and 120 days old, GSE50642 for 40 days old, and GSE27933 for 100 days old, respectively. The expression pattern relative to SALS patients was reported to the far right of the graph. Each point represents the average fold change value of all probe sets representing the gene. As shown in the color bar, *red* indicates upregulation and *green* downregulation. The *blue solid line* represents the regression line and the surrounding *gray area* indicates the 95% confidence interval. Source data for this figure are available on Supplementary Table 1

Deregulation of candidate genes in both human postmortem tissues and terminal stage SOD1^G93A^ mouse samples may be due to reactive changes that occur in final stages of disease, such as cell death and reactive immune responses. To better evaluate whether gene expression changes were a consequence of motor neuron degeneration, we analyzed the expression of drug targets in SOD1^G93A^ mice at different time points (Fig. 3 and Supplementary Figs. 3 and 4). Results obtained showed that, in the majority of cases, their deregulation occurs in animals between 70 and 80 days of age, contestually with the appearance of the first disease symptoms. In three cases (e.g., *Atxn1*, *Fgfr2*, *Tubb5*), gene expression changes started long before symptom onset (40–60 days of age).

### Functional Annotation Clustering of the Most Promising Targets

**Fig. 4:**
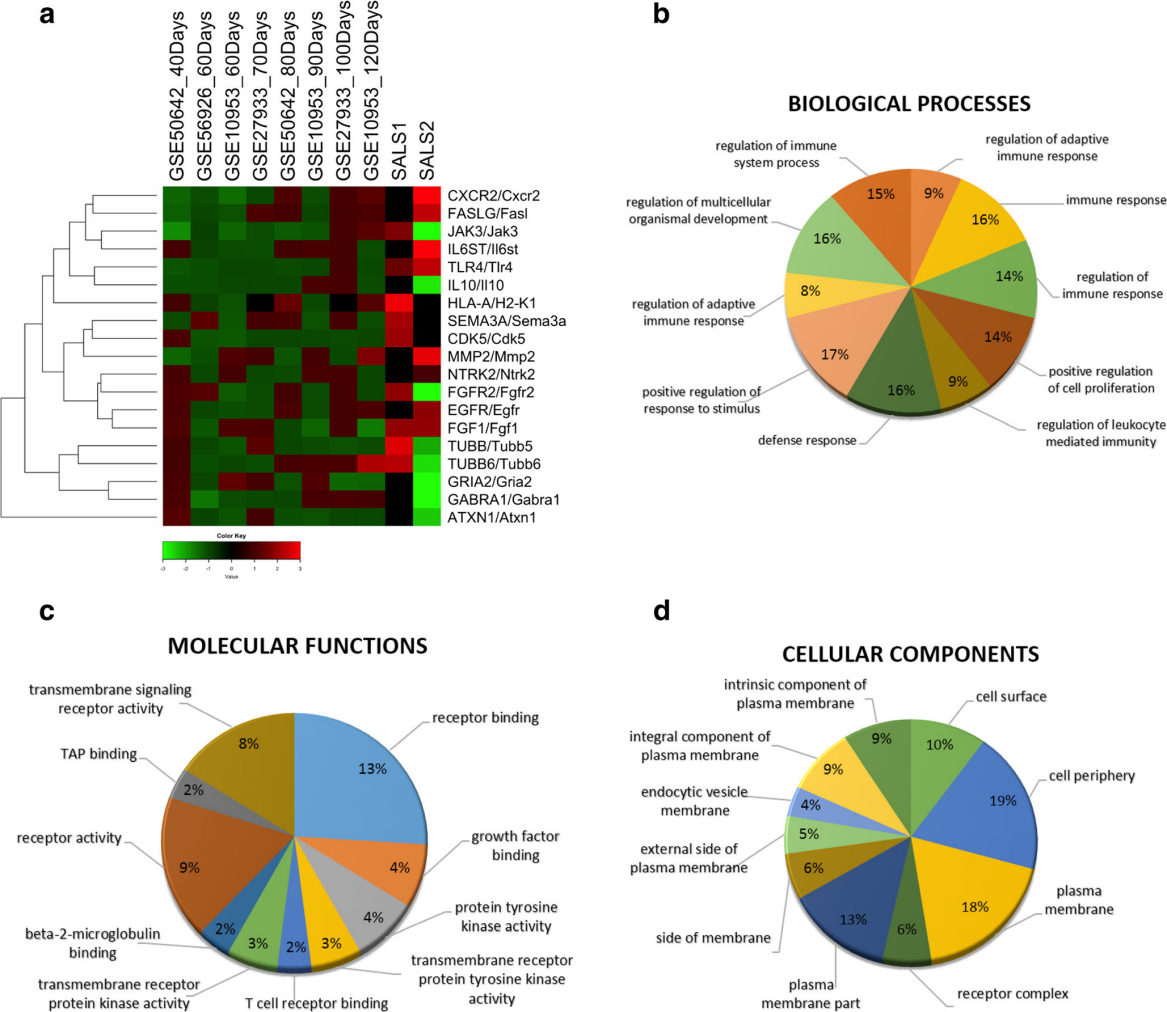
Hierarchical clustering and GO functional enrichment analysis of candidate target genes. **a** Heat map with hierarchical clustering based on Gene Ontology (GO) semantic similarity score of the 19 most promising selected target genes is visualized along with their expression profiles. In this two-dimensional representation, each row represents a single target gene and each column a sample from SOD1^G93A^ mouse models at different stages of disease and SALS patients. Gene symbols for each human/mouse ortholog pair are shown on the *right hand side* of the picture. *Red blocks* represent upregulation and *green blocks* downregulated expression of the relative transcripts, *black* no change. **b–d** Pie charts representing the top 10 enriched (*P* < 0.05) GO terms for the 12 commonly deregulated targets between SALS patients and SOD1^G93A^ mice. The GO terms were subdivided into three GO categories: (b) biological processes, (c) molecular functions, and (d) cellular components. Enrichment analyses were performed using the Enrichment Analysis tool in MetaCore. GO terms or biological features of differently expressed target genes and the percentage of genes represented in each category are indicated. More detailed information are provided in Supplementary Tables 2, 3, and 4

To better understand the biological role of candidate drug targets in ALS pathophysiology, we performed a GO enrichment analysis. The most relevant GO terms were grouped into the three macro-categories: biological processes (BP), molecular functions (MF), and cellular components (CC). The top 10 enriched categories associated with candidate targets are summarized in Fig. 4b–d and mainly included: GO terms for biological processes connected to regulation of adaptive immune response (GO:0002819, *P* = 6.91E^−09^) and positive regulation of cell proliferation (GO:0008284, *P* = 6.91E^−09^), while for molecular functions, the enriched GO terms were receptor binding (GO:0005102, *P* = 4.32E^−07^) and growth factor binding (GO:0019838, *P* = 1.37E^−03^) and, for cellular component, the enriched GO terms were cell surface (GO:0009986, *P* = 2.49E^−06^) and cell periphery (GO:0071944, *P* = 1.25E^−05^) (Supplementary Tables 2, 3, and 4). GO enrichment analysis was also used to define the relationship between target genes and corresponding GO categories and was represented through a hierarchical clustering in which genes were grouped according to their semantic similarities (Fig. 4a).

**Fig. 5:**
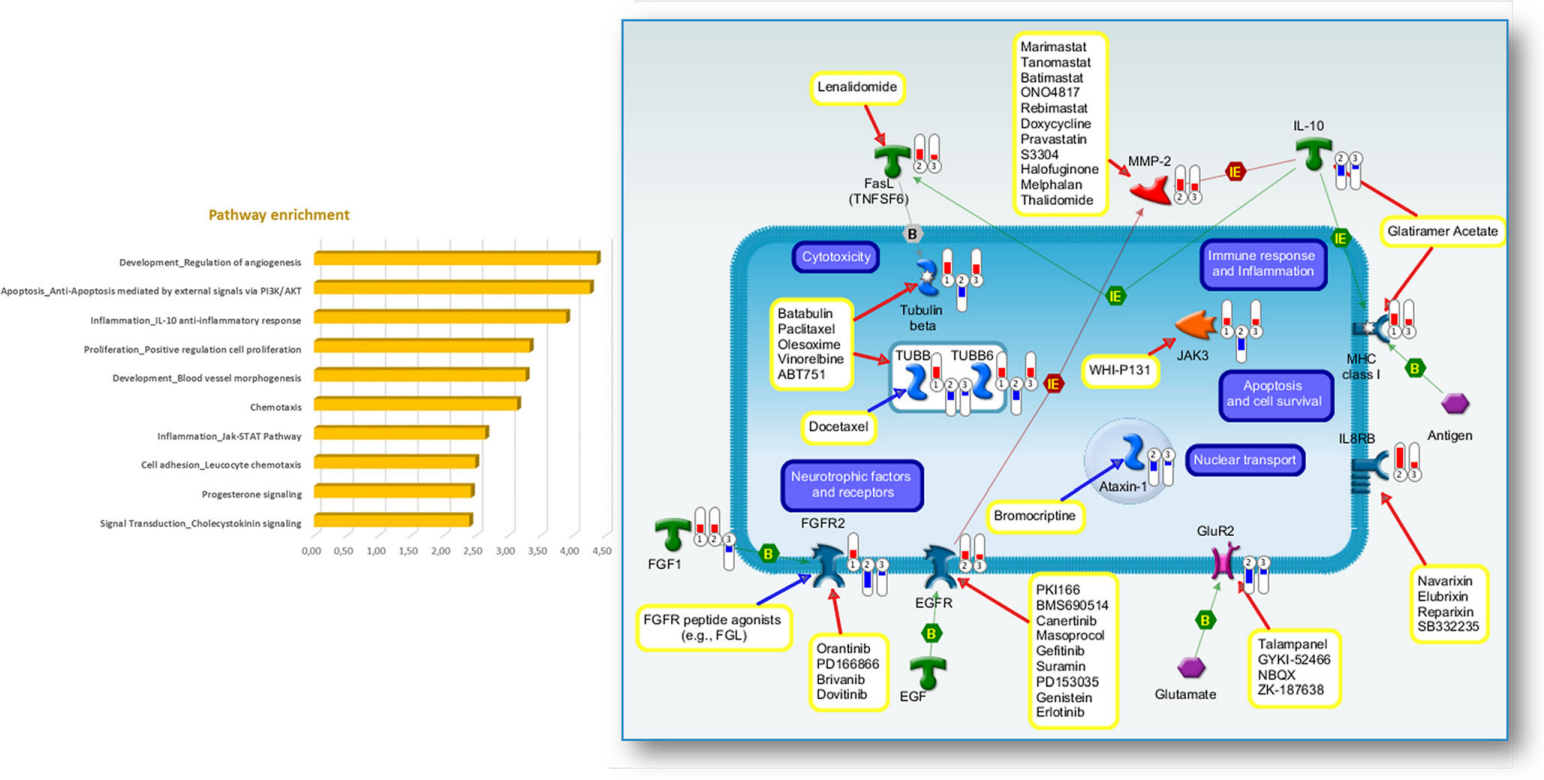
Enrichment analysis for pathway map ontologies revealed significant biological processes associated with the most promising targets commonly deregulated in mouse and human ALS. **a** Representation of the top 10 most significantly enriched (*P* value <0.05) canonical pathway maps associated with the most promising target genes commonly differentially expressed in end-stage SOD1^G93A^ mice and SALS patients when compared with their corresponding controls. A histogram of statistical significance (−log *P* value) is shown: the list is arranged in descending order with the most significant pathways at the *top*. The analysis was performed using the MetaCore™ pathway analysis suite. Detailed information about pathway map enrichment analysis is described in Supplementary Table 5. **b** Interaction pathway map representing the 12 most promising targets deregulated in the same direction in both end-stage SOD1^G93A^ mice and at least one of the two subgroups of SALS patients, together with their corresponding pharmacological modulators. The map was created using the MetaCore Pathway Map Creator tool (GeneGo). Gene expression values are presented on the map as “thermometer-like” figures (*red* for upregulated, *blue* downregulated with thermometer height relative to fold change) with SALS1 patients data represented as thermometer no. 1, SALS2 patient no. 2, and 120-day old SOD1^G93A^ mice no. 3. Pathway objects and links are described separately in the Supplementary Fig. 5

To further investigate the biological significance of promising candidate targets, we performed pathways enrichment analysis by using functional ontologies represented in Metacore repository. This analysis revealed apoptosis, inflammation, immune response, and cell adhesion as the most highly enriched pathways by the candidate drug target list (Fig. 5 and Supplementary Table 5). Based on functional enrichment analysis, we also built a comprehensive pathway map restricted to only direct interactions among the 12 most promising candidate targets and their pharmacological modulators (Fig. 5).

## Discussion

ALS is a progressive and incurable neurodegenerative disease (Pasinelli and Brown 2006; Ferraiuolo et al. 2011). The clinical translational failure of apparently promising preclinical studies conducted in animals may be partly explained by the inadequate nature of these monogenic disease models to mimic the complexity of human pathology (Turner et al. 2001). This underlines the importance to identify common molecular features between animal models and human ALS, allowing the consequent selection and prioritization of the most promising targets for successful therapeutic testing.

In this paper, we choose a meta-analysis approach to compare gene expression profiles, extracted from microarray datasets of SALS patients with those obtained in SOD1^G93A^ mice, in order to select targets that were differentially expressed, in a statistical manner, both in humans and mouse model. To this end, we used the human dataset identified in our previous study (Aronica et al. 2015), restricting the analysis only to genes previously recognized as potential therapeutic targets together their homologs in mouse model (Fig. 2a and Supplementary Table 1) (Ferraiuolo et al. 2007; Maximino et al. 2014; de Oliveira et al. 2013; Saris et al. 2013; Kudo et al. 2010). Results obtained demonstrated that the majority of these drug target genes (12) were statistically deregulated in the same direction in both mice and SALS patients (Fig. 2b, c and Table 2), suggesting that similar molecular mechanisms are shared by both species. Nevertheless, 7/12 genes (*CDK5*, *FGF1*, *GABRA1*, *IL6ST*, *NTRK2(TRKB)*, *SEMA3A*, *TLR4*) were differentially deregulated between mice and SALS patients, representing species-specific mechanisms controlling motor neuron death (Table 2).

In accordance with the aim of this work, we focused the analysis on genes that, being similarly deregulated in human and mouse, are promising candidates for preclinical testing. To investigate the biological role of these drug target genes, we performed an integrated GO and Pathway Maps enrichment analysis that revealed a number of processes and pathways similarly and significantly altered both in affected patients and mice. In particular, the majority of selected targets were involved in common functional pathways, such as immune response, inflammation, cell adhesion, apoptosis, and cell survival, providing additional evidence for the involvement of these processes in the pathophysiology of ALS (Figs. 4 and 5).

In the following paragraphs, we will discuss the 12 most promising candidate drug target genes and highlight their potential relevance for ALS therapy.

### Ataxin-1

Ataxin-1 (*ATXN1* or *SCA1*) encodes a nuclear protein involved in several cellular processes, including protein folding and degradation, mRNA processing, transcriptional regulation, and oxidative stress. ATXN1 normally contains a polyglutamine (polyQ) tract with 22–23 repeats, while the expanded polyQ (23–34 repeats) produces a mutant misfolded protein that tends to form insoluble aggregates into the nucleus, leading to neuronal dysfunction and cell death. In addition to being the cause of spinocerebellar ataxia 1 (SCA1), this gained number of polyQ repeats may also increase genetic risk for ALS (Conforti et al. 2012). In the present study, we found a downregulated expression of *ATXN1* in SALS2 patients and a decreased expression of its mouse homolog (*Atxn1*) in SOD1^G93A^ mice at a presymptomatic stage (~60 days of age) (Figs. 3b and 5). These results are in line with the evidence that the loss of ataxin-1 function leads to neurological alterations in synaptic plasticity and motor deficits through the altered expression of many genes regulated by ATXN1-dependent transcription, including the PolyQ binding protein-1 (*PQBP-1*), dopamine receptor D2 (*DRD2*), RNA polymerase I, and Wnt signaling, all of them also altered in SALS patients (Aronica et al. 2015; Okuda et al. 2003; Matilla et al. 1998; Crespo-Barreto et al. 2010). These data suggest that the alteration of ATXN-1 functions may be involved in the development of both sporadic and familial ALS, in the early stage of disease, making ATXN-1 a promising candidate for disease treatment (Morello and Cavallaro 2015). Among the agents able to prevent aggregation of the mutant ataxin-1 and the consequent neurotoxicity, bromocriptine methylate (BRC), a potent DRD2 agonist approved for the treatment of Parkinson’s disease, showed promising results in ALS preclinical studies (Tanaka et al. 2011; Hearst et al. 2010). Indeed, intraperitoneal administration of BRC in mutant SOD1 mouse confers neuroprotection, sustains motoneuronal functions, slows disease progression, and prolongs survival interval after disease onset, mainly through suppression of oxidative stress-induced cell death. Based on these promising results, this drug is currently being examined in phase IIa clinical trials in patients with ALS (Nagata et al. 2016). Overall, further studies are needed to better deepen the potential therapeutic effects of this and other ATXN-1 pharmacological modulators for ALS treatment (Fig. 5).

### Interleukin-8 Receptor Type B

Chronic inflammation and immune response abnormalities are two processes that may greatly contribute to the progression of ALS disease (Aronica et al. 2015; McCombe and Henderson 2011). A critical component of the inflammatory response is the chemokine (C-X-C motif) receptor 2 (CXCR2), also known as interleukin-8 receptor type B (IL8B), a chemokine receptor involved in microglia activation and neuroinflammatory cascade. Increased expression of *CXCR2* and its rodent homolog (*Cxcr2*) were found, respectively, in SALS2 patients and SOD1^G93A^ mice at a symptomatic stage (80–100 days of age), suggesting that inflammatory chemokine signaling may exert pathogenic effects both in human and mouse ALS (Figs. 3b and 5). These data are corroborated by recent in vitro studies showing that the activation of CXCR2 and its ligand IL-8 induces neurotoxicity and directly contributes to motor neuron degeneration (De Paola et al. 2007). The modulation of CXCR2 may thus represent a potential neuroprotective strategy in ALS therapy. In fact, pharmacological antagonism of CXCR2 produced neuroprotective effects in different animal models of neurodegenerative diseases, including Alzheimer’s, by attenuating chemokine receptor expression in microglia, inhibiting receptor-mediated inflammatory reactivity and enhancing neuronal viability (Marsh and Flemming 2011; Ryu et al. 2015; Gorio et al. 2007). Several pharmacologic agents blocking CXCR2 (e.g., reparixin, navarixin, elubrixin, SB332235) are currently tested in preclinical or clinical trials as anti-inflammatory agents and, among these, reparixin has shown neuroprotective effects in primary cultured motor neurons (Morello and Cavallaro 2015; De Paola et al. 2007). Based on these promising results, we believe that drugs attenuating CXCR2-mediated neurotoxicity may confer neuroprotection against the inflammatory cascade and oxidative damage in ALS pathogenesis (Fig. 5).

### Epidermal Growth Factor Receptor

Epidermal growth factor (EGF) is a potent mitogenic factor that, by activating the EGF receptor tyrosine kinases (EGFR/ErbB), stimulates proliferation, survival, maturation, and migration of a variety of neurons as well as migration, invasion, and proliferation of astrocytes (Kajiwara et al. 2003). Several lines of evidence suggested that the EGFR signaling pathway could play a role in the pathophysiology of many neurodegenerative diseases, including ALS (Offen et al. 2009). In fact, EGFR activation triggers quiescent astrocytes into reactive astrocytes, promoting the neurodegenerative process (Liu et al. 2006). Increased expression of EGFR mRNA observed both in SALS2 patients and SOD1^G93A^ mouse models could thus be related to the reactive astrogliosis surrounding degenerating motor neurons (Figs. 3b and 5) (Vargas and Johnson 2010), and pharmacological inhibition of EGFR signaling cascade may represent a strategy to slow progression of ALS. Treatments with EGFR inhibitors, clinically used in oncology, were reported to be neuroprotective in many models of neuronal injury (Mizuno et al. 2013). In particular, some of these compounds (erlotinib, genistein, masoprocol) have shown positive results in ALS preclinical studies, delaying disease onset (Boston-Howes et al. 2008; Trieu and Uckun 1999). Another important exponent of this drug class, suramin, is currently in preclinical phase for some neurodegenerative disease, like Alzheimer’s and Parkinson’s (Chan and Lin-Shiau 2001; Heneberg 2009). Besides its activity as EGF inhibitor, suramin also contrasts neurotoxicity mediated by nitric oxide synthase (NOS), histone deacetylase (HDAC), and P2Y2 (Xu et al. 2011; Goureau et al. 1993), three proteins that were already extensively implicated in ALS (Janssen et al. 2010; Carlesi et al. 2011). Although the role of EGFR in the pathophysiology of ALS has not been fully clarified, the promising results obtained from the abovementioned and other EGFR inhibitors (e.g., PKI166, BMS690514, canertinib, gefitinib, PD153035) make them attractive candidates for further preclinical studies (Fig. 5).

### Fas Ligand

Fas ligand (FasL or CD95L) is a member of the tumor necrosis factor (TNF) superfamily that activates a cascade several mediators involved in neuroinflammation and apoptotic cell death, such as caspases, p38 kinase and nitric oxide, contributing to mechanisms of neuronal loss and neuritic degeneration. Deregulation of FASL signaling has been associated with the pathogenesis of several neurodegenerative diseases, including ALS (Aronica et al. 2015; Morello and Cavallaro 2015; Raoul et al. 2006; Aebischer et al. 2013; Petri et al. 2006; Cavallaro and Calissano 2006). Indeed, a growing body of data has provided in vivo evidence for the involvement of this cell death pathway in motor neuron death in presymptomatic ALS transgenic mice overexpressing different SOD1 mutations (G37R, G85A, G93A). In accordance with these observations, we found increased expression of the gene encoding FasL in SALS2 patients and SOD1^G93A^ mice on day 70, before the development of motor impairment, suggesting a role of FasL as a possible target for treating ALS in the early stage of the disease (Figs. 3b and 5). A relevant number of therapeutic agents are being developed to interfere with the FasL signaling pathway, conferring neuroprotection against oxidative stress and inflammatory processes. Among these, lenalidomide, a potent immunomodulatory agent that reduces the FasL expression and inactivates downstream effector caspases, has shown to extend the survival of ALS transgenic mice (Neymotin et al. 2009). These data encourage the future clinical evaluation of lenalidomide and other FasL inhibitors as possible therapeutic strategies to prevent or slow ALS disease progression.

### Fibroblast Growth Factor Receptor 2

The fibroblast growth factors (FGFs) constitute a family of multifunctional proteins that mediate various cellular responses, such as neurons and oligodendrocyte proliferation, migration, and differentiation as well as central nervous system development. Their effects are mediated through a subfamily of tyrosine kinase receptors, named FGF receptors (FGFRs). In humans, four FGFRs are described, including FGFR2 subtype. The aberrant activity of FGFs and their receptors, either through gain or loss of function, has been involved in different neuropathological conditions, including ALS. In particular, previous studies showed that FGF-1, released from motor neurons in response to oxidative damage, induces accumulation of its receptors and activation of astrocytes that, in turn, initiate the cascade of events that leads to motor neuron death in ALS (Cassina et al. 2005; Petri et al. 2009; Huang et al. 2012). On the other hand, a significant or complete loss of FGF signaling seems to induce demyelination and axonal damage, contributing to the neurodegenerative and neuroinflammatory processes underlying ALS (Kang et al. 2014). In agreement to these studies, we observed the differential expression of genes encoding FGFs and its receptor FGFR2 in SALS patients (Fig. 5). In addition, a decreased expression of their mouse homologs was found in SOD1^G93A^ transgenic mice, starting on 60–70 days of age and progressing with the advance of disease (Fig. 3b). Pharmacological modulation of FGF/FGFR signaling pathway may thus represent a good therapeutic strategy to contrast neuroinflammatory processes in ALS. The selective FGFR tyrosine kinase inhibitor, PD166866, prevents motor neuron death in ALS mouse models, by reducing astrocyte activation and oxidative damage (Cassina et al. 2005), suggesting the potential therapeutic role of this and other FGF/FGFR signaling inhibitors (orantinib, brivanib, dovitinib, suramin, pentosan polysulfate) in ALS (Fig. 5). Similarly, functional FGFR peptide agonists (e.g., FG Loop (FGL) peptide) seem to be of therapeutic interest for ALS due to their effects not only on neurogenesis but also on synaptic formation, neuron–glia interactions, and inflammation (Woodbury and Ikezu 2014; Ohta et al. 2006; Li et al. 2010; Cox et al. 2013). Further studies are needed to better clarify the involvement of FGF/FGFR signaling in ALS pathophysiology and its role as a future therapeutic target for ALS (Fig. 5).

### AMPA Glutamate Receptor 2

Enhancement of calcium influx through the GluR2 subunit of the α-amino-3-hydroxy-5-methyl-4-isoxazole propionate (AMPA) ionotropic glutamate receptor and the consequent excitotoxicity represent the main plausible hypotheses for selective neuronal death in ALS (Shaw and Ince 1997; Takuma et al. 1999; Kwak and Kawahara 2005). Aberrant expression of GluR2 accelerates the degenerative process of motor neurons and shortens lifespan of ALS transgenic mice (Tateno et al. 2004). Although in SALS2 patients we observed decreased expression of the gene encoding GluR2 subunit (*GRIA2*), its homolog showed a biphasic deregulation in SOD1^G93A^ transgenic mice, being upregulated at 60 days of age (presymptomatic stage) and drastically downregulated later, in parallel to the development of motor deficits (Figs. 3b and 5). This discrepancy may be explained considering that, at the presymptomatic stage, high abundance of GluR2 exposes motor neurons to a greater Ca^2+^ excitotoxicity mediated through AMPA receptors, while downregulation of *GluR2* observed during the symptomatic phase could reflect a transient mechanism of excitotoxicity caused by an imbalance of excitatory–inhibitory synaptic functions that precede motor neuron death (Schutz 2005). Based on these observations, pharmacological strategies aimed to decrease glutamate-mediated neurotoxicity and restore Ca^2+^ homeostasis could be useful for treatment of ALS patients. To this end, the use of some AMPA receptor antagonists (e.g., ZK 187638, NBQX, GYKI-52,466) has been tested and shows promising results in ALS preclinical studies. Indeed, they showed neuroprotective effects by improving motor functions and prolonging the survival of ALS mouse models (Tortarolo et al. 2006; Van Damme et al. 2003; Albo et al. 2004). Unfortunately, another AMPA receptor antagonist, talampanel, which showed promising results in preclinical studies (Paizs et al. 2011), was ineffective when tested in patients (Pascuzzi et al. 2010). Differential expression of AMPA receptor subunits between the two subgroups of SALS patients, as demonstrated previously (Aronica et al. 2015), may contribute to explain this clinical failure. In light of this evidence, further investigations are needed to verify the potential clinical benefits of talampanel and other AMPA receptor antagonists for the personalized treatment of ALS patients (Fig. 5).

### Major Histocompatibility Complex Class I

Deregulation of the antigen processing and presentation pathways is considered a possible cause of the harmful non-autonomous cell toxicity occurring during ALS pathology. Among the main mediators of this inflammatory signaling cascade, of great relevance are the molecules belonging to the major histocompatibility complex (MHC) class I, a large family of proteins encoded by a complex of genes, such as *HLA-A*, *HLA-B*, and *HLA-C*. In particular, an increased expression of these proteins and their murine homologs has been found in the spinal cord of ALS patients and microglia surrounding motor neurons of presymptomatic ALS animal models, respectively (Lampson et al. 1990; Graber et al. 2010; Casas et al. 2013). In line with these findings, we found upregulated expression of genes encoding MHC class I molecules, particularly *HLA-A*, in the motor cortex of SALS1 patients and a similar expression was observed in samples from SOD1^G93A^ transgenic mouse models at presymptomatic stages (70–80 days of age) (Figs. 3a and 5). All these data contribute to highlight the role of MHC class I molecules in increasing ALS susceptibility, supporting their possible role as targets for limiting neurodegeneration in this disease (Fig. 5). Glatiramer acetate (GA) is an immunomodulator agent able to reduce the MHC class I expression as well as bind to the neuronal and glial MHC molecules expressed in the central nervous system and influencing communication between such cells. This drug, clinically used to treat multiple sclerosis, was reported to exert a neuroprotective role in several neurological conditions, including ALS (Scorisa et al. 2011). Administration of GA, in fact, delays disease onset and progression in ALS animal models by decreasing neuroinflammatory processes and contributing to reduce the loss of synaptic inputs to motor neurons occurring during the disease (Habisch et al. 2007). Phase II clinical trials for GA are currently conducted in ALS patients (Gordon et al. 2006).

### Interleukin-10

Interleukin 10 (IL-10) is a potent anti-inflammatory cytokine involved in mechanisms of immunomodulation and maintaining the anti-inflammatory environment within the central nervous system. There is a strong body of evidence demonstrating the neuroprotective role of this factor against motor neuron injury in ALS (Xin et al. 2011). Accordingly, downregulation of the gene encoding IL-10 was observed in SALS2 patients, while the expression of its homolog in SOD1^G93A^ mice was stable but with lowest values as compared to their relative littermate controls (Figs. 3b and 5). Based on these observations, pharmacological modulation of IL-10 signaling may represent a promising strategy for the treatment of ALS. To this regard, it is interesting to note that glatiramer acetate, besides its activity as MHC modulator (discussed above), exerts its anti-inflammatory effects also by inducing T helper cells to secrete IL-10 and other anti-inflammatory cytokines, providing a multi-targeted mechanism of action for the treatment of ALS (Arnon and Aharoni 2004; Vieira et al. 2003).

### Janus Kinase 3

The family of Janus kinases comprises four tyrosine kinases (JAK1, JAK2, JAK3, and TYK2) which together with the family of signal transducer and activator of transcription (STAT) plays key roles in numerous cytokine-mediated signaling pathways. Deregulation of this signaling pathway has been widely associated with numerous neuroinflammatory and neurodegenerative diseases, including ALS (Aronica et al. 2015; Nicolas et al. 2013). In particular, differential expression of genes encoding JAKs has been found in the motor cortex of SALS patients (Fig. 5). In addition, the expression of *Jak3* in SOD1^G93A^ mice was found to be low in the first 60 days of age but then increased with the disease progression and paralysis development, peaking on day 120, just before death (Fig. 3a). These results imply the blockade of JAK-STAT signaling and, in particular, those of JAK3 activity, as a promising therapeutic strategy to contrast the degenerative progression of ALS, especially for patient subtypes associated with aberrant activation of this pathway. In support of this theory, several studies showed neuroprotective effects of JAK inhibitors (Ignarro et al. 2013). Among these, WHI-P131 treatment exhibited promising results in ALS preclinical studies, slowing disease progression and increasing survival of ALS transgenic mice (Trieu et al. 2000). Further studies are still necessary to confirm the therapeutic potential of WHI-P131 and other JAK inhibitors (e.g., AG490, AZD1480) in ALS therapy (Fig. 5).

### Matrix Metalloproteinase 2

Matrix metalloproteinases (MMPs) are a large family of calcium-dependent zinc-containing endopeptidases exerting important functions in several cellular processes, including synaptic remodeling, degradation of the extracellular matrix, and neuronal regeneration (Singh et al. 2015). In agreement to these evidences, deregulation of MMP system is thought to contribute strongly to the development of many neurodegenerative diseases, including ALS (Brkic et al. 2015; Niebroj-Dobosz et al. 2010). The upregulation of different MMP-mRNAs, such as *MMP-2*, both in SALS2 patients as well as in SOD1^G93A^ mice, supports the role of MMP-signaling cascade as a promising therapeutic target for ALS (Figs. 3b and 5). Indeed, pharmacological inhibition of MMP expression, synthesis, and activity has shown beneficial effects in various neurodegenerative conditions (Jarvelainen et al. 2009). Among the agents able to reduce MMP2 levels, particular attention should be given to the drug thalidomide, a potent anti-inflammatory agent that has shown promising results both in preclinical and clinical ALS studies (Kiaei et al. 2006; Stommel et al. 2009). Despite its use as therapeutic agent for ALS treatment, however, it presents several important side effects. To overcome this limit, other MMP inhibitors (e.g., marimastat, tanomastat, batimastat, ONO4817, doxycycline, rebimastat, pravastatin, S3304, halofuginone, melphalan) have been developed and many of these, currently tested in preclinical or clinical trials as therapeutic agents in oncology, have also demonstrated therapeutic effects for several neurodegenerative diseases, such as Alzheimer’s (Costa et al. 2011; Dong et al. 2009). These encouraging results make the MMP inhibition a possible therapeutic strategy to treat ALS disease (Fig. 5).

### Tubulin Proteins

Among the main components of the cytoskeleton are microtubules, cytoskeletal filaments composed of heterodimers, including α/β-tubulin. Tubulin beta proteins (TUBBs) are highly expressed in neurons where they play an important role both in neurogenesis and development as well as axon guidance and maintenance. Recent evidence indicates that disruption of tubulin beta system in motor neurons, through oxidative damage, may affect their interaction with other important proteins, such as dynein, interfering with the stability and function of microtubules (Millecamps and Julien 2013). In particular, aberrant expression and aggregation of TUBB proteins were found in the spinal cord of mutant SOD1^G93A^ mice before the onset of symptoms (Lederer et al. 2007a; Kabuta et al. 2009). In accordance with these results, we observed the differential expression of genes encoding TUBB1/TUBB6 in SALS patients and the altered mRNA expression of their murine homologs (*Tubb5* and *Tubb6*) in SOD1^G93A^ mice at a presymptomatic stage. These data suggest a potential causative role of TUBB system in the early events of ALS progression (Figs. 3 and 5). Pharmacological approaches aimed to functional recovery of tubulin system might contribute to restore the axonal transport defects and altered nerve signals in ALS. This hypothesis is sustained by several studies that highlight as taxanes (paclitaxel, docetaxel) and other microtubule-targeting drugs (batabulin, vinorelbine, ABT751), mainly used in oncology treatments, also show neuroprotective effects. Indeed, they restore impaired transmission of nerve impulses in many neurodegenerative diseases, including Alzheimer’s (Shemesh and Spira 2011). Despite their clinical efficacy, these drugs may induce neurotoxic damages and, thus, several other compounds have been developed to prevent side effects. One representative agent of these new drug class is olesoxime, a neuroprotective and reparative compound, targeting tubulins, which reduces astrogliosis and microglial activation, inducing neurite outgrowth and improving nerve cell remyelination, promotes axonal sprouting, and prevents the activation of the mitochondrial apoptotic pathway, delaying disease onset and improving survival in ALS animal models (Sunyach et al. 2012; Bordet and Pruss 2009). Although ALS phase II/III clinical trials with olesoxime showed disappointing results, this failure may be due to the lack of a molecular-based recruitment of ALS patients in specific subgroups, as well as to a problem of intervening time between starting drug treatment and the stage of the disease, rather than an absence of its therapeutic efficacy (Martin 2010; Lenglet et al. 2014). Further studies are needed to evaluate the potential therapeutic efficacy of olesoxime and other related compounds in the early phases of ALS progression.

## Conclusions

One of the major problems associated with the development of effective ALS treatments is the inadequacy of the animal models (Turner et al. 2001). Indeed, despite the wide use of mouse models for ALS in biomedical research, they would mimic only partial aspects of human pathology, often leading to a consistent lack of concordance between the preclinical and clinical studies. Although the role of some potential candidate targets in ALS pathogenesis has been studied in detail in individual studies, no integrated analysis has previously elucidated which potential drug targets are consistently deregulated in both human and animal pathology. Therefore, a better understanding of the similarities and differences between the animal and human ALS pathophysiology is of fundamental importance to the rational identification of therapeutic interventions capable of stopping or at least effectively modifying the course of ALS.

Here, we used a meta-analysis approach to identify potential drug targets commonly altered both in mouse and human ALS, to optimize future preclinical investigations. Our interspecies comparison demonstrated that candidate target genes in mouse models exhibit expression patterns similar those of the human conditions. In the same way, more pathways related to these target genes were commonly deregulated, revealing the existence of a conserved transcriptional signature underlying ALS neurodegeneration.

In addition to identify new potential pharmacological targets, our analysis proposes to offer a rational approach for “drug repositioning.” In fact, in light of the emerging molecular taxonomy of SALS patients, many known drugs that were abandoned at clinical stages because of their low efficacy and/or toxicity may be reevaluated for their potential therapeutic role, with the consequent possibility to reduce both the time and costs associated with drug discovery and development.

Despite our remarkable efforts in data collection, processing, and integration using advanced statistical analysis and data visualization tools, a limitation of the present study is the relatively small number of datasets (nine) used. Since these datasets have been generated by different microarray platforms, species, disease stages, conditions, and tissue types, it may be hard to identify reliable disease-specific target genes. Finally, data obtained in the murine model may not be able to accurately recapitulate the extremely complex processes underlying human pathology. In fact, although the SOD1^G93A^ transgenic mouse has been a bedrock for ALS research and the most important and well-used model for well over a decade, it manifests abnormal phenotypic features that might arise from extreme SOD1 protein overexpression rather than being the effect of the SOD1^G93A^ mutation itself and motor neuron death generally occurs over short periods of time. This limits the subsequent translation of the results to the human condition, where symptoms do not emerge for several years and the disease develops through a combination of multiple factors that are beyond genetic mutations and currently not captured by any model.

Nevertheless, our study represents an important contribution in the field of ALS, not only supporting the utility of SOD1^G93A^ mice as animal models for both familial and sporadic ALS but also contributing to the selection of potential candidates for the development of more accurate preclinical studies and their effective translation into patient-oriented clinical trials. In addition, it is interesting to note that deregulated expression of the majority of candidate targets was detected at onset of the disease, offering the possibility to propose them for an early and more effective diagnosis and therapy.

To our knowledge, this is the first meta-analysis-based study that identifies drug target candidates by comparing expression changes between mouse models and human ALS. We believe that our findings could be considered as starting point for future investigations for the development of more effective therapeutic strategies for this devastating disease.

## Electronic supplementary material

Supplementary Fig. 1 Meta-analysis of gene expression profiles of SOD1^G93A^ mice and SALS1 patients reveals common candidate therapeutic targets. **a** Hierarchical clustering heat map visualization of the gene expression pattern for 33 genes encoding promising therapeutic targets across different datasets from SOD1^G93A^ mice (at different ages and stages of disease) and SALS1 patients. **b** Hierarchical clustering of nine statistically significant (*P* value <0.05) differentially expressed target genes commonly deregulated in both end-stage SOD1^G93A^ mice and SALS1 patients. In both two-dimensional presentations, rows represent target genes and columns denote datasets used in our meta-analysis: from the left to right, these included expression profiles of motor neurons from spinal cord of SOD1^G^ ^93A^ mice at 40, 60, 70, 80, 90, 100, and 120-day old and motor neurons from motor cortex of SALS1 patients. Gene symbols for each human/mouse ortholog pair are shown on the *right hand side* of the picture. Genes were clustered using a hierarchical clustering based on Euclidean distances of average fold change values represented in a linear scale with complete linkage method as parameter. In the dendrograms shown (*left*), the length and the subdivision of the branches display the relatedness of the expression of the genes. The fold change values were calculated as the ratio between SALS1 patients versus individual controls for the human dataset and between SOD1^G93A^ mice versus littermate control groups for each murine dataset. As shown in the color bar, *red* indicates upregulation, *green* downregulation, and *black* no change. Complete gene list and corresponding fold change values are found in Supplementary Table 1. (PDF 334 kb)

Supplementary Fig. 2 Meta-analysis of gene expression profiles of SOD1^G93A^ mice and SALS2 patients reveals common candidate therapeutic targets. **a** Hierarchical clustering heat map visualization of the gene expression pattern of 54 genes encoding promising therapeutic targets across different datasets from SOD1^G93A^ mice (at different ages and stages of disease) and SALS2 patients. **b** Hierarchical clustering of 16 statistically significant (*P* value <0.05) differentially expressed target genes commonly deregulated in both end-stage SOD1^G93A^ mice and SALS2 patients. In both two-dimensional presentations, rows represent target genes and columns denote datasets used in our meta-analysis: from the left to right, these included expression profiles of motor neurons from spinal cord of SOD1^G93A^ mice at 40, 60, 70, 80, 90, 100, and 120-day old and motor neurons from motor cortex of SALS2 patients. Gene symbols for each human/mouse ortholog pair are shown on the *right hand side* of the picture. Genes were clustered using a hierarchical clustering based on Euclidean distances of average fold change 38 values represented in a linear scale with complete linkage method as parameter. In the dendrograms shown (*left*), the length and the subdivision of the branches display the relatedness of the expression of the genes. The fold change values were calculated as the ratio between SALS2 patients versus individual controls for the human dataset and between SOD1^G93A^ mice versus littermate control groups for each murine dataset. As shown in the color bar, *red* indicates upregulation, *green* downregulation, and *black* no change. Complete gene list and corresponding fold change values are found in Supplementary Table 1. (PDF 445 kb)

Supplementary Fig. 3 Time-course analysis of nine statistically significant differentially expressed target genes commonly deregulated in SALS1 patients and SOD1^G93A^ mice at different stages of disease. For each panel is shown the correlation of the expression fold changes (*y*-axis) of target genes among SALS1 patients versus individual controls and SOD1^G93A^ mice versus littermate controls at 40, 60, 100, and 120 days of age (*x*-axis). For generating time-course analysis of SOD1^G93A^ mice, we referred to following datasets: GSE10953 for 60, 90, and 120 days old, GSE50642 for 40 days old, and GSE27933 for 100 days old, respectively. The expression pattern relative to SALS1 patients was reported to the far right of the graph. Each point represents the average fold change value of all probe sets representing the gene. As shown in the color bar, *red* indicates upregulation and *green* downregulation. The *blue solid line* represents the regression line and the surrounding *gray area* indicates the 95% confidence interval. Source data for this figure are available on the Supplementary Table 1. (PDF 167 kb)

Supplementary Fig. 4 Time-course analysis of 16 statistically significant differentially expressed candidate target genes commonly deregulated in SALS2 patients and SOD1^G93A^ mice at different stages of disease. For each panel is shown the correlation of the expression fold changes (*y*-axis) of target genes among SALS2 patients versus individual controls and SOD1^G93A^ mice versus littermate controls at 40, 60, 100, and 120 days of age (*x*-axis). For generating time-course analysis of SOD1^G93A^ mice, we referred to following datasets: GSE10953 for 60, 90 and 120 days old, GSE50642 for 40 days old and GSE27933 for 100 days old respectively. The expression pattern relative to SALS2 patients was reported to the far right of the graph. Each point represents the average fold change value of all probe sets representing the gene. As shown in the color bar, *red* indicates upregulation and *green* downregulation. The *blue solid line* represents the regression line and the surrounding *gray area* indicates the 95% confidence interval. Source data for this figure are available on the Supplementary Table 1. (PDF 260 kb)

Supplementary Fig. 5 Legend describing symbols used in the MetaCore pathway map. (PDF 337 kb)

Supplementary Table 1 Comparison of gene expression changes between SALS patients and SOD1^G93A^ mice (at different ages and stages of disease) for the 70 potential candidate targets for ALS. (PDF 77 kb)

Supplementary Table 2 The 10 most significantly enriched (*P* value <0.05) biological processes according to Gene Ontology. (PDF 10 kb)

Supplementary Table 3 The 10 most significantly enriched (*P* value <0.05) molecular functions according to Gene Ontology. (PDF 10 kb)

Supplementary Table 4 The 10 most significantly enriched (*P* value <0.05) cellular components according to Gene Ontology. (PDF 10 kb)

Supplementary Table 5 List of the top 10 pathway maps enriched (*P* value <0.05) based on MetaCore repository (PDF 10 kb)
