# Supplementary figures and images for: Selection and Prioritization of Candidate Drug Targets for Amyotrophic Lateral Sclerosis Through a Meta-Analysis Approach
Source: J Mol Neurosci. 2017 Feb 24;61(4):563–80. doi: 10.1007/s12031-017-0898-9 (PMC5359376; doi:10.1007/s12031-017-0898-9)

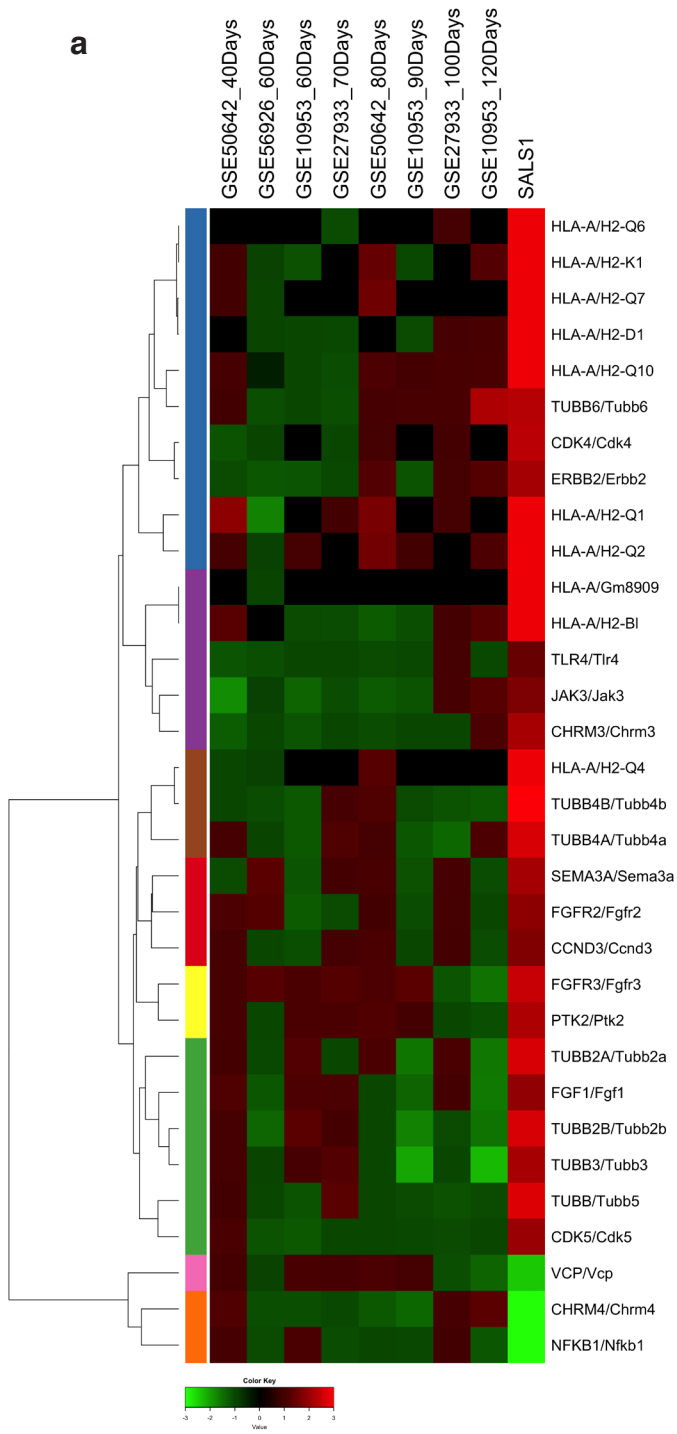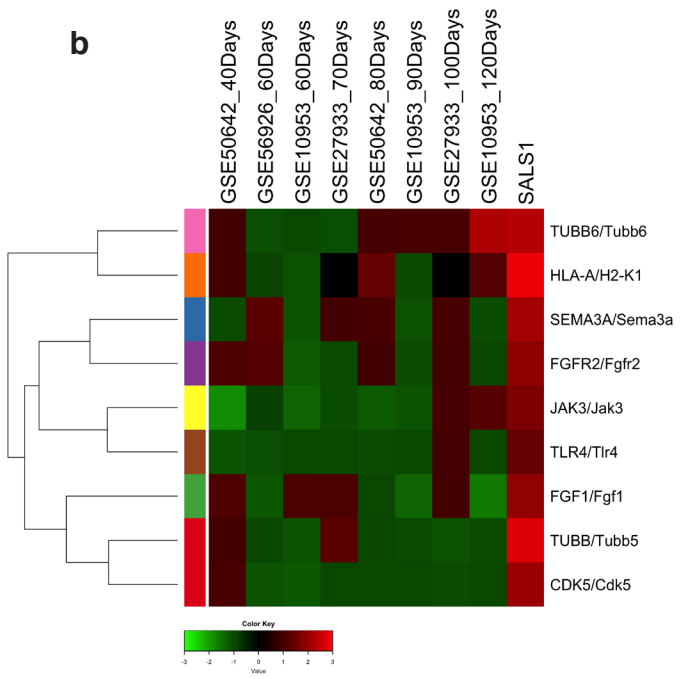

Supplement: Supplementary file 1 — Meta-analysis of gene expression profiles of SOD1G93A mice and SALS1 patients reveals common candidate therapeutic targets. a Hierarchical clustering heat map visualization of the gene expression pattern for 33 genes encoding promising therapeutic targets across different datasets from SOD1G93A mice (at different ages and stages of disease) and SALS1 patients. b Hierarchical clustering of nine statistically significant (P value <0.05) differentially expressed target genes commonly deregulated in both end-stage SOD1G93A mice and SALS1 patients. In both two-dimensional presentations, rows represent target genes and columns denote datasets used in our meta-analysis: from the left to right, these included expression profiles of motor neurons from spinal cord of SOD1G 93A mice at 40, 60, 70, 80, 90, 100, and 120-day old and motor neurons from motor cortex of SALS1 patients. Gene symbols for each human/mouse ortholog pair are shown on the right hand side of the picture. Genes were clustered using a hierarchical clustering based on Euclidean distances of average fold change values represented in a linear scale with complete linkage method as parameter. In the dendrograms shown (left), the length and the subdivision of the branches display the relatedness of the expression of the genes. The fold change values were calculated as the ratio between SALS1 patients versus individual controls for the human dataset and between SOD1G93A mice versus littermate control groups for each murine dataset. As shown in the color bar, red indicates upregulation, green downregulation, and black no change. Complete gene list and corresponding fold change values are found in Supplementary Table 1. (PDF 334 kb) [file 12031_2017_898_MOESM1_ESM.pdf]

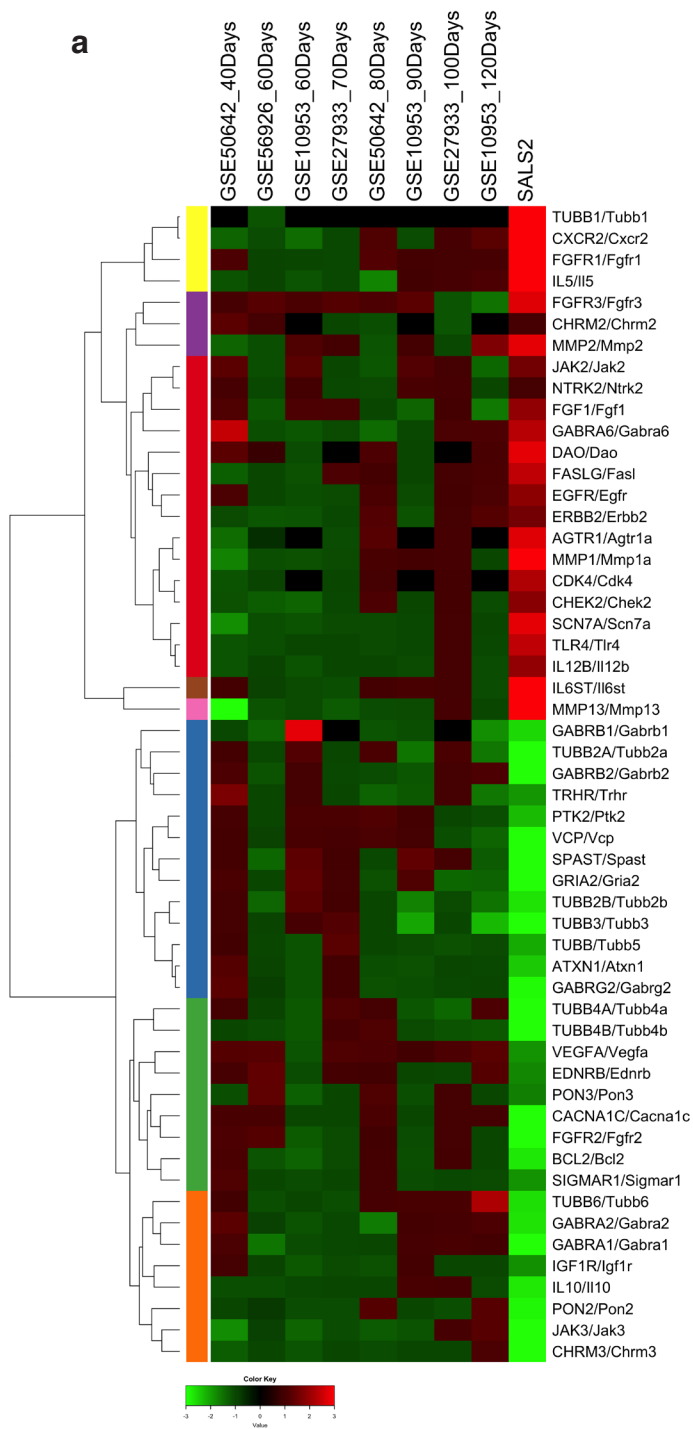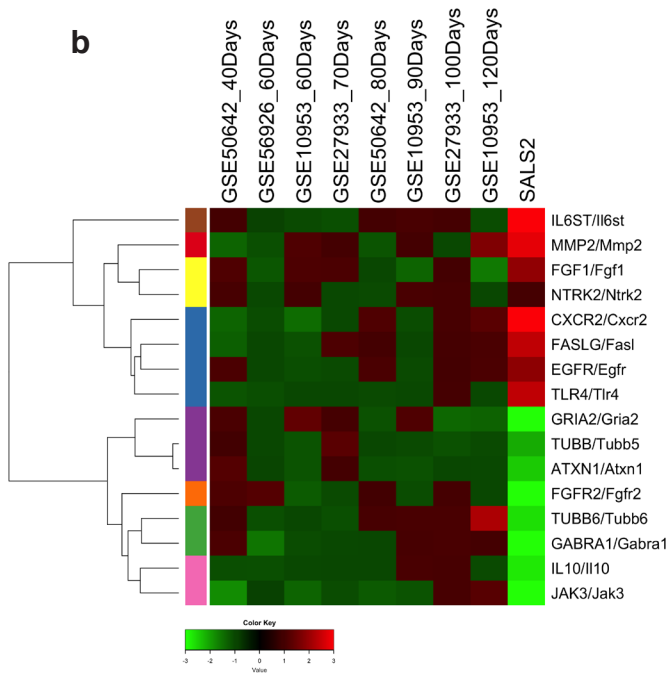

Supplement: Supplementary file 2 — Meta-analysis of gene expression profiles of SOD1G93A mice and SALS2 patients reveals common candidate therapeutic targets. a Hierarchical clustering heat map visualization of the gene expression pattern of 54 genes encoding promising therapeutic targets across different datasets from SOD1G93A mice (at different ages and stages of disease) and SALS2 patients. b Hierarchical clustering of 16 statistically significant (P value <0.05) differentially expressed target genes commonly deregulated in both end-stage SOD1G93A mice and SALS2 patients. In both two-dimensional presentations, rows represent target genes and columns denote datasets used in our meta-analysis: from the left to right, these included expression profiles of motor neurons from spinal cord of SOD1G93A mice at 40, 60, 70, 80, 90, 100, and 120-day old and motor neurons from motor cortex of SALS2 patients. Gene symbols for each human/mouse ortholog pair are shown on the right hand side of the picture. Genes were clustered using a hierarchical clustering based on Euclidean distances of average fold change 38 values represented in a linear scale with complete linkage method as parameter. In the dendrograms shown (left), the length and the subdivision of the branches display the relatedness of the expression of the genes. The fold change values were calculated as the ratio between SALS2 patients versus individual controls for the human dataset and between SOD1G93A mice versus littermate control groups for each murine dataset. As shown in the color bar, red indicates upregulation, green downregulation, and black no change. Complete gene list and corresponding fold change values are found in Supplementary Table 1. (PDF 445 kb) [file 12031_2017_898_MOESM2_ESM.pdf]

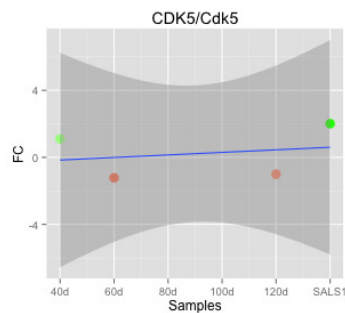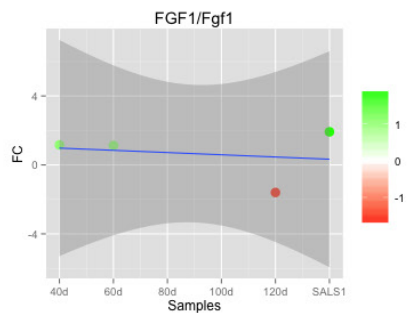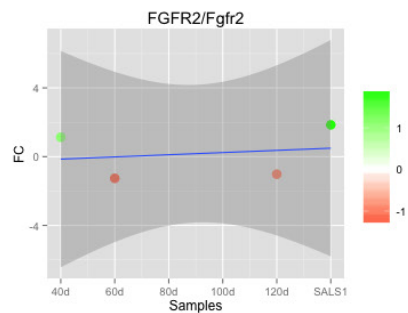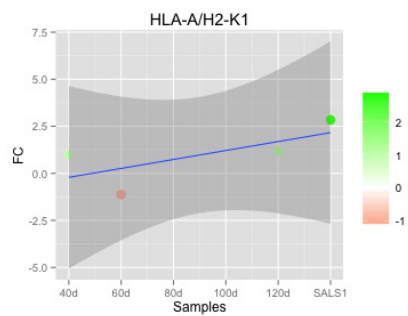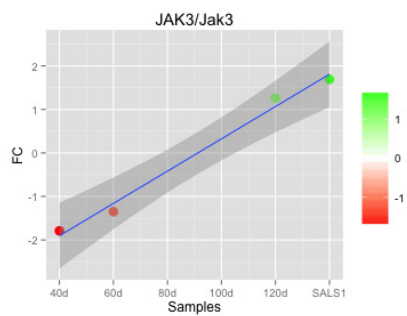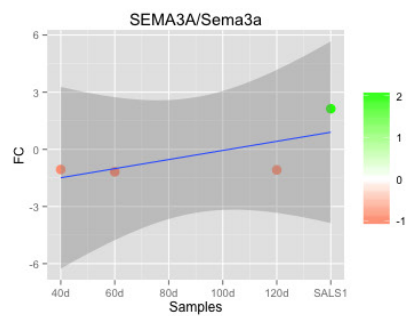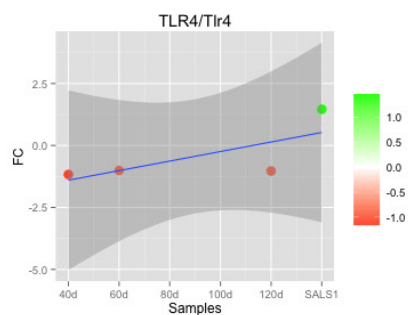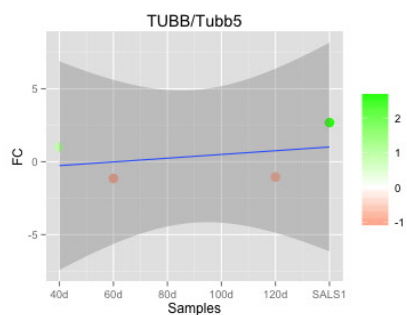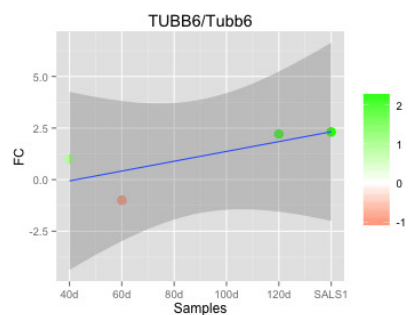

Supplement: Supplementary file 3 — Time-course analysis of nine statistically significant differentially expressed target genes commonly deregulated in SALS1 patients and SOD1G93A mice at different stages of disease. For each panel is shown the correlation of the expression fold changes (y-axis) of target genes among SALS1 patients versus individual controls and SOD1G93A mice versus littermate controls at 40, 60, 100, and 120 days of age (x-axis). For generating time-course analysis of SOD1G93A mice, we referred to following datasets: GSE10953 for 60, 90, and 120 days old, GSE50642 for 40 days old, and GSE27933 for 100 days old, respectively. The expression pattern relative to SALS1 patients was reported to the far right of the graph. Each point represents the average fold change value of all probe sets representing the gene. As shown in the color bar, red indicates upregulation and green downregulation. The blue solid line represents the regression line and the surrounding gray area indicates the 95% confidence interval. Source data for this figure are available on the Supplementary Table 1. (PDF 167 kb) [file 12031_2017_898_MOESM3_ESM.pdf]

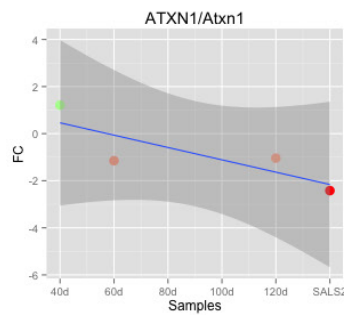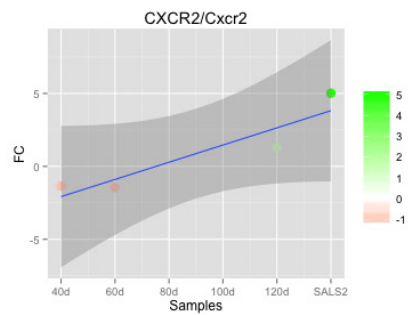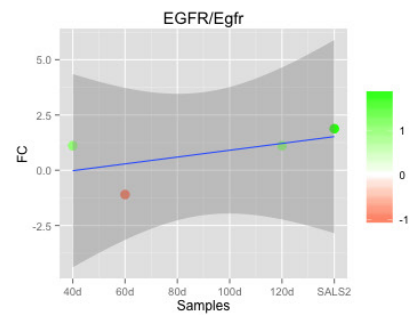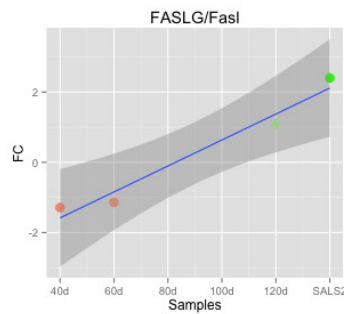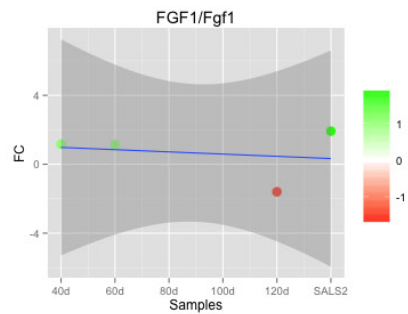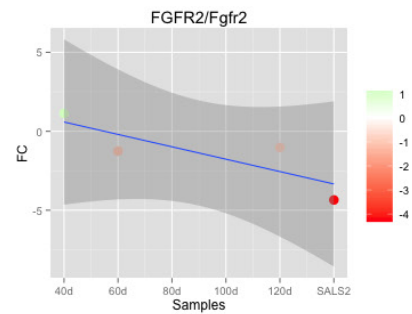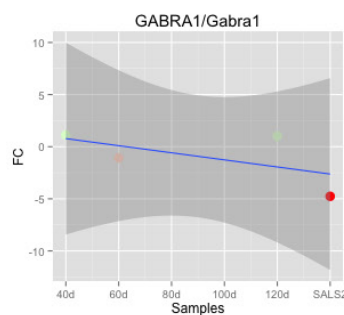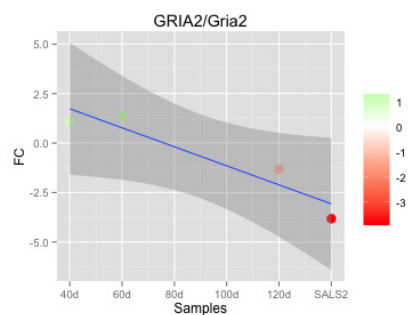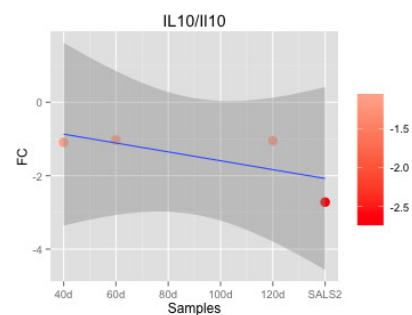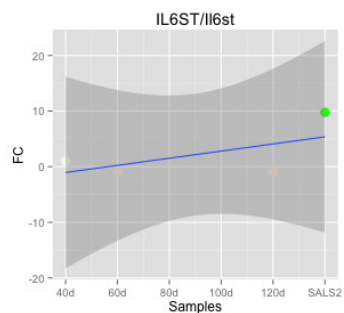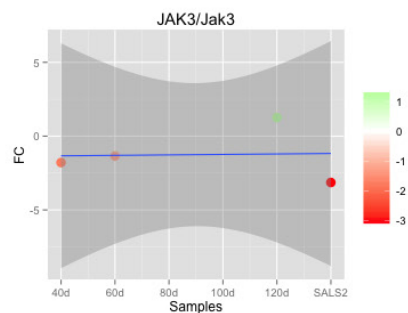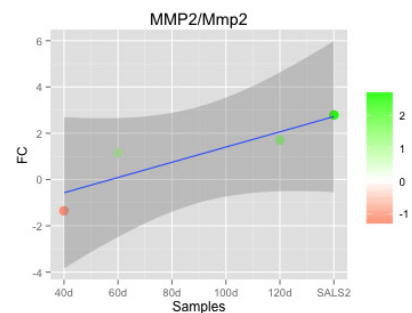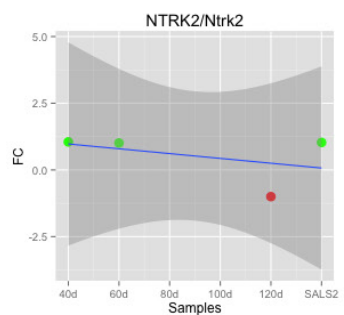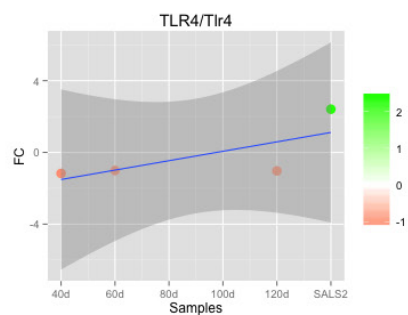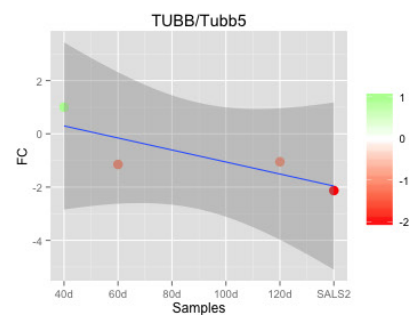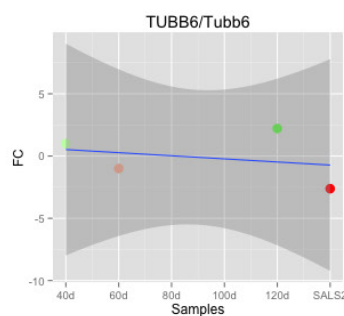

Supplement: Supplementary file 4 — Time-course analysis of 16 statistically significant differentially expressed candidate target genes commonly deregulated in SALS2 patients and SOD1G93A mice at different stages of disease. For each panel is shown the correlation of the expression fold changes (y-axis) of target genes among SALS2 patients versus individual controls and SOD1G93A mice versus littermate controls at 40, 60, 100, and 120 days of age (x-axis). For generating time-course analysis of SOD1G93A mice, we referred to following datasets: GSE10953 for 60, 90 and 120 days old, GSE50642 for 40 days old and GSE27933 for 100 days old respectively. The expression pattern relative to SALS2 patients was reported to the far right of the graph. Each point represents the average fold change value of all probe sets representing the gene. As shown in the color bar, red indicates upregulation and green downregulation. The blue solid line represents the regression line and the surrounding gray area indicates the 95% confidence interval. Source data for this figure are available on the Supplementary Table 1. (PDF 260 kb) [file 12031_2017_898_MOESM4_ESM.pdf]
