# Supplementary material for: Selection and Prioritization of Candidate Drug Targets for Amyotrophic Lateral Sclerosis Through a Meta-Analysis Approach
Source: J Mol Neurosci. 2017 Feb 24;61(4):563–80. doi: 10.1007/s12031-017-0898-9 (PMC5359376; doi:10.1007/s12031-017-0898-9)
Supplement: Supplementary file 5 — Legend describing symbols used in the MetaCore pathway map. (PDF 337 kb) [file 12031_2017_898_MOESM5_ESM.pdf]

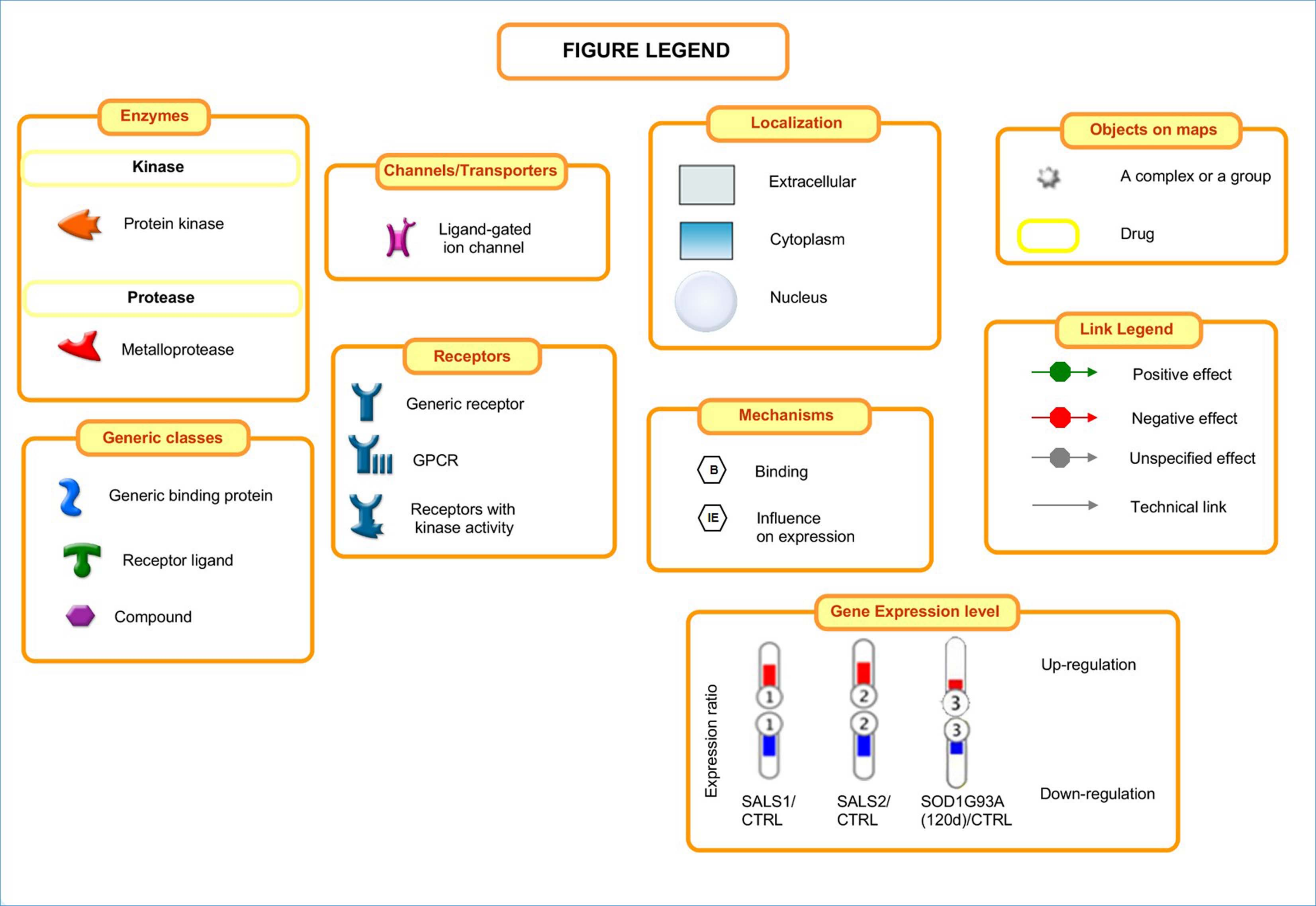

FIGURE LEGEND
Enzymes
Kinase
Protein kinase
Protease
Metalloprotease
Channels/Transporters
Ligand-gated ion channel
Localization
Extracellular
Cytoplasm
Nucleus
Objects on maps
A complex or a group
Drug
Receptors
Generic receptor
GPCR
Receptors with kinase activity
Mechanisms
B
Binding
IE
Influence on expression
Link Legend
Positive effect
Negative effect
Unspecified effect
Technical link
Generic classes
Generic binding protein
Receptor ligand
Compound
Gene Expression level
Expression ratio
1
1
2
2
3
3
SALS1/ CTRL
SALS2/ CTRL
SOD1G93A (120d)/CTRL
Up-regulation
Down-regulation
