# Supplementary material for: Selection and Prioritization of Candidate Drug Targets for Amyotrophic Lateral Sclerosis Through a Meta-Analysis Approach
Source: J Mol Neurosci. 2017 Feb 24;61(4):563–80. doi: 10.1007/s12031-017-0898-9 (PMC5359376; doi:10.1007/s12031-017-0898-9)
Supplement: Supplementary file 6 — Comparison of gene expression changes between SALS patients and SOD1G93A mice (at different ages and stages of disease) for the 70 potential candidate targets for ALS. (PDF 77 kb) [file 12031_2017_898_MOESM6_ESM.pdf]

Supplementary Table 1. Comparison of gene expression changes between SALS patients and SOD1G93A mice (at different ages and stages of disease) for the 70 potential candidate targets for ALS.

Gene ID (human)	GeneSymbol Human/Mouse	SOD1G93A vs control 40 days (GSE50642)	SOD1G93A vs control 60 days (GSE56926)	SOD1G93A vs control 60 days (GSE10953)	SOD1G93A vs control 70 days (GSE27933)	SOD1G93A vs control 80 days (GSE50642)	SOD1G93A vs control 90 days (GSE10953)	SOD1G93A vs control 100 days (GSE27933)	SOD1G93A vs control 120 days (GSE10953)	SALS1 /Controls	SALS2 /Controls
185	AGTR1/Agtr1a	-1,45	-0,63	-	-1,07	1,23	-	1,06	-	-	2,72
6310	ATXN1/Atxn1	1,21	-0,9	-1,15	1,01	-1,11	-1,14	-1,01	-1,04	-	-2,42
596	BCL2/Bcl2	1,09	-1,18	-1,34	-1,05	1,02	-1,12	1,02	-1	-	-2,71
775	CACNA1C/Cacna1c	1,07	1,07	-1,02	-1	1,09	-1	1,03	1,04	-	-3,27
896	CCND3/Ccnd3	1,03	-1,01	-1,11	1,03	1,09	-1,01	1,02	-1,08	1,73	-
1019	CDK4/Cdk4	-1,16	-0,97	-	-1,02	1,04	-	1,01	-	2,36	2,25
1020	CDK5/Cdk5	1,1	-1,16	-1,22	-1,01	-1	-1,04	-1,05	-1,01	2,01	-
11200	CHEK2/Chek2	-1,14	-1,27	-1,35	-1,04	1,13	-1,01	1,02	-1,07	-	1,84
1129	CHRM2/Chrm2	1,31	1,03	-	-1,02	-1,1	-	-1,18	-	-	1,05
1131	CHRM3/Chrm3	-1,27	-1,02	-1,18	-1,01	-1,07	-1	-1,01	1,11	2,17	-3,1
1132	CHRM4/Chrm4	1,18	-1,1	-1,09	-1,04	-1,22	-1,39	1,06	1,3	-3,72	-
3579	CXCR2/Cxcr2	-1,36	-1,07	-1,45	-1,04	1,15	-1,07	1,06	1,28	-	5,02
1610	DAO/Dao	1,32	0,79	-1,07	-	1,13	-1,02	-	1,08	-	2,79
1910	EDNRB/Ednrb	1,06	1,37	-1,08	1,02	1	-1,02	-1,04	1,21	-	-1,76
1956	EGFR/Egfr	1,11	-1,01	-1,1	-1,05	1,1	-1,05	1,02	1,11	-	1,88
2064	ERBB2/Erbb2	-1,06	-1,2	-1,18	-1,04	1,2	-1,16	1,04	1,21	2,12	1,6
356	FASLG/Fasl	-1,29	-1	-1,14	1,14	1,01	-1,02	1,03	1,09	-	2,4
2246	FGF1/Fgf1	1,16	-1,19	1,13	1,12	-1,02	-1,36	1,02	-1,6	1,92	1,92
2260	FGFR1/Fgfr1	1,14	-0,91	-1,02	-1,04	1,19	1,02	1,03	1,06	-	4,72
2263	FGFR2/Fgfr2	1,13	1,21	-1,26	-1,05	1	-1,07	1,03	-1,02	1,85	-4,35
2261	FGFR3/Fgfr3	1,05	1,23	1,09	1,22	1,14	1,29	-1,17	-1,51	2,49	2,72
2554	GABRA1/Gabra1	1,12	-1,55	-1,07	-1,03	-1,01	1,05	1,07	1,02	-	-4,76
2555	GABRA2/Gabra2	1,32	-0,86	-1,16	-1,04	-1,61	1,02	1,05	1,13	-	-2,66
2559	GABRA6/Gabra6	2,46	-1,12	-1,19	-1,05	-1,43	-1,03	1,11	1,13	-	2,33
2560	GABRB1/Gabrb1	-1,03	-1,33	2,76	-	-1,17	-1,12	-	-1,82	-	-2,56
2561	GABRB2/Gabrb2	1,13	-1,15	1,03	-1,03	-1,08	-1,2	1,04	1,14	-	-3,34
2566	GABRG2/Gabrg2	1,3	-0,81	-1,15	1,01	-1,13	-1,1	-1,04	-1,02	-	-2,88
2891	GRIA2/Gria2	1,09	-1	1,38	1,04	-1,14	1,16	-1,39	-1,33	-	-3,82
3105	HLA-A/H2-Q1	1,85	-1,7	-	1	1,63	-	1,04	-	2,84	-
3105	HLA-A/Gm8909	-	-0,98	-	-	-	-	-	-	2,84	-
3105	HLA-A/H2-Q4	-1,01	-0,86	-	-	1,25	-	-	-	2,84	-
3105	HLA-A/H2-Q6	-	-	-	-1,05	-	-	1,03	-	2,84	-
3105	HLA-A/H2-Q7	1	-0,96	-	-	1,53	-	-	-	2,84	-
3105	HLA-A/H2-Q2	1,03	-0,85	1,04	-	1,57	1	-	1,13	2,84	-
3105	HLA-A/H2-K1	1	-0,87	-1,13	-	1,42	-1,03	-	1,19	2,84	-
3105	HLA-A/H2-D1	-	-0,92	-1,01	-1,03	-	-1,05	1,05	1,08	2,84	-
3105	HLA-A/H2-Bl	1,23	-	-1,06	-1,07	-1,26	-1,12	1,02	1,26	2,84	-
3105	HLA-A/H2-Q10	1,06	-0,42	-1,01	-1,07	1,14	1,01	1,08	1,1	2,84	-
3480	IGF1R/Igf1r	1,04	-0,91	-1,22	-1,05	-1,13	1,02	-1,01	-1,01	-	-1,84
3586	IL10/Il10	-1,09	-1,11	-1,03	-1,02	-1,02	1,07	1,05	-1,05	-	-2,72
3593	IL12B/Il12b	-1,17	-0,99	-1,16	-1,01	-1,02	-1,06	1,03	-1,07	-	1,93
3567	IL5/Il5	-1,15	-0,93	-1,18	-1,04	-1,72	1	1,05	1,14	-	6,89
3572	IL6ST/Il6st	1,02	-0,87	-1,06	-1,11	1,02	1,08	1,04	-1,08	-	9,76
3717	JAK2/Jak2	1,3	-1,12	1,28	-1,02	-1,2	1,2	1,04	-1,39	-	1,57
3718	JAK3/Jak3	-1,79	-0,85	-1,35	-1,07	-1,24	-1,15	1,05	1,26	1,69	-3,14
4312	MMP1/Mmp1a	-1,69	-1,1	-1,14	-1,05	1,08	1,01	1,06	-1,02	-	3,1
4322	MMP13/Mmp13	-4,65	-1,15	-1,06	-1,24	-1,08	-1,06	1,05	-1	-	7,53
4313	MMP2/Mmp2	-1,35	-1,11	1,15	1,01	-1,17	1,01	-1,03	1,71	-	2,79
4790	NFKB1/Nfkb1	1,04	-1,05	1,09	-1,07	-1	-1,03	1	-1,2	-6,45	-
4915	NTRK2/Ntrk2	1,05	-1,03	1,01	-1,04	-1,06	1,07	1,06	-1	-	1,03
5445	PON2/Pon2	-1,01	-0,76	-1,08	-1,07	1,21	-1,02	-1,09	1,25	-	-2,84
5446	PON3/Pon3	-1,12	1,35	-1,32	-1	1,15	-1,12	1,04	-1,02	-	-1,67
5747	PTK2/Ptk2	1,06	-1,02	1,09	1,09	1,17	1,02	-1,02	-1,09	2,21	-2,28
6332	SCN7A/Scn7a	-1,82	-1,1	-1,16	-1,05	-1,06	-1,04	1,04	-1,02	-	2,78
10371	SEMA3A/Sema3a	-1,06	1,29	-1,18	1,02	1,08	-1,13	1,06	-1,08	2,13	-
10280	SIGMAR1/Sigmar1	1,16	-1	-1,07	-1,02	1,04	-1,07	-1,04	-1,05	-	-1,85
6683	SPAST/Spast	1,02	-1,4	1,34	1,02	-1,03	1,37	1,03	-1,23	-	-3,43
7099	TLR4/Tlr4	-1,17	-1,09	-1,01	-1,01	-1,06	-1,03	1,04	-1,03	1,46	2,41
7201	TRHR/Trhr	1,65	-1	1,03	-1,01	-1,33	-1,22	1,04	-1,58	-	-1,9
203068	TUBB/Tubb5	1	-1,02	-1,15	1,27	-1,01	-1,06	-1,14	-1,05	2,68	-2,13
81027	TUBB1/Tubb1	-	-1,15	-	-	-	-	-	-	-	4,98
7280	TUBB2A/Tubb2a	1,01	-1,04	1,19	-1,01	1,09	-1,56	1,1	-1,58	2,62	-3,8
347733	TUBB2B/Tubb2b	1,03	-1,38	1,3	1,02	-1,02	-1,7	-1,06	-1,54	2,62	-2,66
10381	TUBB3/Tubb3	1,04	-0,92	1,05	1,2	-1	-2,08	-1,02	-2,27	2,14	-3,41

10382	TUBB4A/Tubb4a	1,04	-0,95	-1,21	1,15	1,04	-1,2	-1,39	1,14	2,62	-3,85
10383	TUBB4B/Tubb4b	-1,01	-1,07	-1,22	1,05	1,15	-1,06	-1,15	-1,21	3,04	-4,14
84617	TUBB6/Tubb6	1	-1,12	-1	-1,12	1,06	1,08	1,07	2,21	2,31	-2,62
7415	VCP/Vcp	1,01	-0,88	1,08	1,05	1,12	1,03	-1,11	-1,36	-2,4	-3,62
7422	VEGFA/Vegfa	1,22	1,23	-1,17	1,16	1,13	1	1,14	1,27	-	-1,87

For each gene, average fold change values of differential expression between SALS patients versus individual controls and SOD1-G93A transgenic mouse versus littermate controls, are shown. For all SOD1-G93A mice studies, DataSet Accession ID and age in days at which the gene was found differentially expressed, were reported. In the last two columns are reported fold change values referred to both subgroups of SALS patients.

Abbreviations: *AGTR1* (angiotensin II receptor, type 1); *ATXN1* (ataxin 1); *BCL2* (B-cell CLL/lymphoma 2); *CACNA1C* (calcium channel, voltage-dependent, L type, alpha 1C subunit); *CCND3* (cyclin D3); *CDK4* (cyclin-dependent kinase 4); *CDK5* (cyclin-dependent kinase 5); *CHEK2* (checkpoint kinase 2); *CHRM2* (cholinergic receptor, muscarinic 2); *CHRM3* (cholinergic receptor, muscarinic 2); *CHRM4* (cholinergic receptor, muscarinic 2); *CXCR2* (chemokine (C-X-C motif) receptor 2); *DAO* (D-amino-acid oxidase); *EDNRB* (endothelin receptor type B); *EGFR* (epidermal growth factor receptor); *ERBB2* (erb-b2 receptor tyrosine kinase 2); *FASLG* (Fas ligand (TNF superfamily, member 6)); *FGF1* (fibroblast growth factor 1 (acidic)); *FGFR1* (fibroblast growth factor receptor 1); *FGFR2* (fibroblast growth factor receptor 2); *FGFR3* (fibroblast growth factor receptor 3); *GABRA1* (gamma-aminobutyric acid (GABA) A receptor, alpha 1); *GABRA2* (gamma-aminobutyric acid (GABA) A receptor, alpha 2); *GABRA6* (gamma-aminobutyric acid (GABA) A receptor, alpha 6); *GABRB1* (gamma-aminobutyric acid (GABA) A receptor, beta 1); *GABRB2* (gamma-aminobutyric acid (GABA) A receptor, beta 2); *GABRG2* (gamma-aminobutyric acid (GABA) A receptor, gamma 2); *GRIA2* (glutamate receptor, ionotropic, AMPA 2); *HLA-A* (major histocompatibility complex, class I, A); *IGF1R* (insulin like growth factor 1 receptor); *IL10* (interleukin 10); *IL12B* (interleukin 12B); *IL5* (interleukin 5); *IL6ST* (interleukin 6 signal transducer); *JAK2* (Janus kinase 2); *JAK3* (Janus kinase 3); *MMP1* (matrix metallopeptidase 1); *MMP1 3* (matrix metallopeptidase 1 3); MMP2 (matrix metallopeptidase 2); *NFKB1* (nuclear factor of kappa light polypeptide gene enhancer in B-cells 1); *NTRK2* (neurotrophic tyrosine kinase, receptor, type 2); *PON2* (paraoxonase 2); *PON3* (paraoxonase 3); *PTK2* (protein tyrosine kinase 2); *SCN7A* (sodium channel, voltage gated, type VII alpha subunit); *SEMA3A* (sema domain, immunoglobulin domain (Ig), short basic domain, secreted, (semaphorin) 3A); *SIGMAR1* (sigma non-opioid intracellular receptor 1); *SPAST* (spastin); *TLR4* (toll-like receptor 4); *TRHR* (thyrotropin-releasing hormone receptor); *TUBB* (tubulin, beta class I); *TUBB1* (tubulin, beta 1 class VI); *TUBB2A* (tubulin, beta 2A class IIa); *TUBB2B* (tubulin, beta 2A class IIb); *TUBB3* (tubulin, beta 3 class III); *TUBB4A* (tubulin, beta 4A class IVa); *TUBB4B* (tubulin, beta 4A class IVb); *TUBB6* (tubulin, beta 6 class V); *VCP* (valosin containing protein); *VEGFA* (vascular endothelial growth factor A).
