# Supplementary material for: Selection and Prioritization of Candidate Drug Targets for Amyotrophic Lateral Sclerosis Through a Meta-Analysis Approach
Source: J Mol Neurosci. 2017 Feb 24;61(4):563–80. doi: 10.1007/s12031-017-0898-9 (PMC5359376; doi:10.1007/s12031-017-0898-9)
Supplement: Supplementary file 7 — The 10 most significantly enriched (P value <0.05) biological processes according to Gene Ontology. (PDF 10 kb) [file 12031_2017_898_MOESM7_ESM.pdf]

Supplementary Table 2. The 10 most significantly enriched (P value < 0.05) biological processes according to Gene Ontology.

GO_Biological Processes	Ratio	-LOG(pValue)	p Value*	FDR
GO:0002819:regulation of adaptive immune response	9/223	11,629	2,35E-12	6,91E-09
GO:0006955:immune response	16/1761	11,327	4,71E-12	6,91E-09
GO:0050776:regulation of immune response	14/1185	11,177	6,66E-12	6,91E-09
GO:0008284:positive regulation of cell proliferation	14/1212	11,045	9,02E-12	6,91E-09
GO:0002703:regulation of leukocyte mediated immunity	9/265	10,955	1,11E-11	6,91E-09
GO:0006952:defense response	16/2018	10,425	3,76E-11	1,75E-08
GO:0048584:positive regulation of response to stimulus	17/2421	10,406	3,92E-11	1,75E-08
GO:0002822:regulation of adaptive immune response based on somatic recombination of immune receptors built from immunoglobulin superfamily	8/206	10,189	6,47E-11	2,52E-08
GO:2000026:regulation of multicellular organismal development	16/2210	9,828	1,49E-10	5,15E-08
GO:0002682:regulation of immune system process	15/1931	9,505	3,13E-10	8,87E-08

*P-Values have been obtained through Hypergeometric analysis and corrected by FDR method.
