# Supplementary material for: Selection and Prioritization of Candidate Drug Targets for Amyotrophic Lateral Sclerosis Through a Meta-Analysis Approach
Source: J Mol Neurosci. 2017 Feb 24;61(4):563–80. doi: 10.1007/s12031-017-0898-9 (PMC5359376; doi:10.1007/s12031-017-0898-9)
Supplement: Supplementary file 8 — The 10 most significantly enriched (P value <0.05) molecular functions according to Gene Ontology. (PDF 10 kb) [file 12031_2017_898_MOESM8_ESM.pdf]

Supplementary Table 3. The 10 most significantly enriched (P value < 0.05) molecular functions according to Gene Ontology.

GO_Molecular Functions	Ratio	-LOG(pValue)	p Value*	FDR
GO:0005102:receptor binding	13/1808	8,701	1,99E-09	4,32E-07
GO:0019838:growth factor binding	4/149	4,833	1,47E-05	1,37E-03
GO:0004713:protein tyrosine kinase activity	4/149	4,722	1,90E-05	1,37E-03
GO:0004714:transmembrane receptor protein tyrosine kinase activity	3/79	4,154	7,01E-05	3,80E-03
GO:0042608:T cell receptor binding	2/16	3,923	1,19E-04	5,18E-03
GO:0019199:transmembrane receptor protein kinase activity	3/103	3,812	1,54E-04	5,48E-03
GO:0030881:beta-2-microglobulin binding	2/22	3,64	2,29E-04	5,48E-03
GO:0004872:receptor activity	9/2314	3,61	2,46E-04	5,48E-03
GO:0046977:TAP binding	2/23	3,61	2,51E-04	5,48E-03
GO:0004888:transmembrane signaling receptor activity	8/1833	3,543	2,87E-04	5,48E-03

*P-Values have been obtained through Hypergeometric analysis and corrected by FDR method.
