# Supplementary material for: Selection and Prioritization of Candidate Drug Targets for Amyotrophic Lateral Sclerosis Through a Meta-Analysis Approach
Source: J Mol Neurosci. 2017 Feb 24;61(4):563–80. doi: 10.1007/s12031-017-0898-9 (PMC5359376; doi:10.1007/s12031-017-0898-9)
Supplement: Supplementary file 9 — The 10 most significantly enriched (P value <0.05) cellular components according to Gene Ontology. (PDF 10 kb) [file 12031_2017_898_MOESM9_ESM.pdf]

Supplementary Table 4. The 10 most significantly enriched (P value < 0.05) cellular components according to Gene Ontology.

GO_Cellular Components	Ratio	-LOG(pValue)	p Value*	FDR
GO:0009986:cell surface	10/917	7,96	1,10E-08	2,49E-06
GO:0071944:cell periphery	19/6026	6,958	1,10E-07	1,25E-05
GO:0005886:plasma membrane	18/5909	6,167	6,81E-07	5,16E-05
GO:0043235:receptor complex	6/337	6,014	9,69E-07	5,50E-05
GO:0044459:plasma membrane part	13/2955	5,805	1,57E-06	7,11E-05
GO:0098552:side of membrane	6/541	4,833	1,47E-05	5,56E-04
GO:0009897:external side of plasma membrane	5/326	4,744	1,80E-05	5,85E-04
GO:0030666:endocytic vesicle membrane	4/171	4,572	2,68E-05	7,59E-04
GO:0005887:integral component of plasma membrane	9/1786	4,369	4,27E-05	1,08E-03
GO:0031226:intrinsic component of plasma membrane	9/1861	4,229	5,91E-05	1,34E-04

*P-Values have been obtained through Hypergeometric analysis and corrected by FDR method.
