# Supplementary material for: Selection and Prioritization of Candidate Drug Targets for Amyotrophic Lateral Sclerosis Through a Meta-Analysis Approach
Source: J Mol Neurosci. 2017 Feb 24;61(4):563–80. doi: 10.1007/s12031-017-0898-9 (PMC5359376; doi:10.1007/s12031-017-0898-9)
Supplement: Supplementary file 10 — List of the top 10 pathway maps enriched (P value <0.05) based on MetaCore repository (PDF 10 kb) [file 12031_2017_898_MOESM10_ESM.pdf]

**Supplementary Table 5.** List of the top 10 pathway maps enriched based on Metacore analysis.

| Pathway Maps | RATIO | -LOG (pValue) | p Value | FDR |
|---|---|---|---|---|
| Development_Regulation of angiogenesis | 6/223 | 4,37 | 4,25E-05 | 2,58E-03 |
| Apoptosis_Anti-Apoptosis mediated by external signals via PI3K/AKT | 6/223 | 4,27 | 5,43E-05 | 2,58E-03 |
| Inflammation_IL-10 anti-inflammatory response | 3/87 | 3,90 | 1,27E-04 | 4,02E-03 |
| Proliferation_Positive regulation cell proliferation | 5/221 | 3,33 | 4,64E-04 | 1,02E-02 |
| Development_Blood vessel morphogenesis | 5/228 | 3,27 | 5,35E-04 | 1,02E-02 |
| Chemotaxis | 4/137 | 3,14 | 7,24E-04 | 1,15E-02 |
| Inflammation_Jak-STAT Pathway | 4/186 | 2,65 | 2,25E-03 | 3,06E-02 |
| Cell adhesion_Leucocyte chemotaxis | 4/205 | 2,49 | 3,21E-03 | 3,79E-02 |
| Progesterone signaling | 4/214 | 2,43 | 3,75E-03 | 3,79E-02 |
| Signal Transduction_Cholecystokinin signaling | 3/106 | 2,40 | 3,98E-03 | 3,79E-02 |

*P-Values have been obtained through Hypergeometric analysis and corrected by FDR method.
